# Structural Insight into and Mutational Analysis of Family 11 Xylanases: Implications for Mechanisms of Higher pH Catalytic Adaptation

**DOI:** 10.1371/journal.pone.0132834

**Published:** 2015-07-10

**Authors:** Wenqin Bai, Cheng Zhou, Yueju Zhao, Qinhong Wang, Yanhe Ma

**Affiliations:** 1 National Engineering Laboratory for Industrial Enzymes, Tianjin Institute of Industrial Biotechnology, Chinese Academy of Sciences, Tianjin, 300308, China; 2 National Engineering Laboratory for Industrial Enzymes, Institute of Microbiology, Chinese Academy of Sciences, Beijing, 100101, China; 3 School of Life Science, Shanxi Normal University, Linfen, 041004, China; 4 Institute of Agro-Products Processing Science and Technology, Chinese Academy of Agriculture Sciences, Beijing, 100193, China; Russian Academy of Sciences, Institute for Biological Instrumentation, RUSSIAN FEDERATION

## Abstract

To understand the molecular basis of higher pH catalytic adaptation of family 11 xylanases, we compared the structures of alkaline, neutral, and acidic active xylanases and analyzed mutants of xylanase Xyn11A-LC from alkalophilic *Bacillus* sp. SN5. It was revealed that alkaline active xylanases have increased charged residue content, an increased ratio of negatively to positively charged residues, and decreased Ser, Thr, and Tyr residue content relative to non-alkaline active counterparts. Between strands β6 and β7, alkaline xylanases substitute an α-helix for a coil or turn found in their non-alkaline counterparts. Compared with non-alkaline xylanases, alkaline active enzymes have an inserted stretch of seven amino acids rich in charged residues, which may be beneficial for xylanase function in alkaline conditions. Positively charged residues on the molecular surface and ionic bonds may play important roles in higher pH catalytic adaptation of family 11 xylanases. By structure comparison, sequence alignment and mutational analysis, six amino acids (Glu16, Trp18, Asn44, Leu46, Arg48, and Ser187, numbering based on Xyn11A-LC) adjacent to the acid/base catalyst were found to be responsible for xylanase function in higher pH conditions. Our results will contribute to understanding the molecular mechanisms of higher pH catalytic adaptation in family 11 xylanases and engineering xylanases to suit industrial applications.

## Introduction

Xylanase (EC 3.2.1.8) can hydrolyze β-1,4-xylosidic linkages of xylan, a major component of hemicelluloses in plant cell walls [1,2]. Based on hydrophobic cluster analysis and sequence homology of the catalytic domain, xylanases are mainly classified into glycoside hydrolase (GH) families 10 and 11 [3], which have a (α/β)_8_-barrel fold [4] and a β-jelly roll structure [5], respectively. Xylanase has potential economic environmentally friendly applications [6]. A major application of xylanases is in the paper and pulp industry, in which the enzyme can reduce the consumption of toxic chlorine-containing chemicals and improve the pulp brightness [7]. Because of their small molecular mass (about 20 kDa) and cellulase-free activity, family 11 xylanases can easily penetrate cellulose fiber networks without damaging the fibers and are therefore suitable for the pulp bleaching process [8].

Xylanases used in the paper industry are required to be stable and active at high temperature and alkaline pH [9]. However, most xylanases reported to date are active and stable at mesophilic temperatures and neutral or weakly acidic pH. Investigators have carried out much research on the thermophilicity of family 11 xylanases, which can contribute to improving the thermophilicity of mesophilic enzymes for industrial applications [10–16]. However, little is known about the molecular adaptation of family 11 xylanases to alkaline conditions. Such adaptations are complex and the limited studies to date on high pH adaptation of proteins [4,17–20] are not sufficient to draw universal conclusions on the adaptation mechanisms. However, those findings provide clues for research into higher pH catalytic adaptation of family 11 xylanases. Many crystal structures of family 11 xylanases have been determined, which provide material for structural insights into higher pH catalytic adaptation of the enzyme.

In this paper, we collected structural information of all family 11 mesophilic xylanases with known pH-dependent activity. By structural comparison of acidophilic, neutral, and alkaline enzymes, molecular features of higher pH catalytic activity of family 11 xylanases were systematically identified. We have previously characterized a family 11 xylanase, Xyn11A-LC from alkalophilic *Bacillus* sp. SN5, that exhibited maximal activity at pH 7.5–8.0 and 55°C [21]. We purified and crystallized this enzyme and solved the structure at 1.49 Å resolution [22]. Here, site-directed mutagenesis of Xyn11A-LC was performed to further investigate the molecular mechanism of higher pH catalytic adaptation of family 11 xylanases. These results enhance the understanding of molecular mechanisms of higher pH catalytic adaptation of family 11 xylanases, and may facilitate engineering of the enzyme to be active in higher pH conditions for industrial applications.

## Methods

### Phylogenetic analysis

The characterized family 11 xylanases were obtained from the website http://www.cazy.org/GH11_characterized.html. All mesophilic xylanases reported in literature with known pH-dependent activity were selected from the characterized xylanases. Amino acid sequences of their catalytic domains were retrieved from the GenBank database. Multiple sequence alignment of the amino acid sequences was performed using the ClustalW program [23]. Phylogenetic trees were constructed using MEGA 5.05 [24] with the neighbor-joining method [25] and the minimum-evolution method [26] in the p-distance and Poisson models. Bootstrap values were calculated based on 1,000 replications of the data.

### Structural comparison with other family 11 xylanases

The total amino acid compositions of the catalytic domains were calculated using online tools http://www.expasy.org/tools/protparam.html. The independent-samples T test was performed using SPSS 16.0. The coordinates of thirteen family 11 mesophilic xylanases with known pH-dependent activity were extracted from the Protein Data Bank (PDB). The surface residues that have ≥30% accessible surface areas were identified in Swiss-PDB Viewer [27] using the default parameters. The hydrogen bond content was calculated with DSSP [28]. Structure-based alignment was performed using ENDscript [29] and ESPript [30]. Three-dimensional structures of all xylanases were superimposed with the PyMOL program [31]. Salt bridges were calculated online by submitting the coordinate files to http://bioinformatica.isa.cnr.it/ESBRI/; salt bridges were assigned when the distance between the two atoms of opposite charge was less than 4 Å. In all calculations, water molecules and hetero-atoms were excluded from the coordinate files. Mutant structures were modeled using Discovery Studio software. Structure visualization was performed using PyMOL software.

### Mutant construction

Site-directed mutagenesis was performed by PCR of whole plasmid using primers containing a mutant codon. The recombinant plasmid pET28a-Xyn11A-LC containing the xylanase gene *Xyn11A-LC* was used as the template. The mutagenesis PCR products were transformed into *Escherchia coli* BL21 (DE3) for expression after *Dpn*I digestion. The variant genes were confirmed by DNA sequencing.

### Expression and purification of the wild-type protein and mutants

The expression and purification of the wild-type and mutant proteins was performed following the same procedure as described previously [21]. The purity of the proteins was analyzed by 12% SDS-PAGE. The concentration of the proteins was determined with a protein assay kit (Bio-Rad, Hercules, CA, USA). Purified enzyme was stored at 2 mg/mL at −80°C in 20 mM Tris/HCl (pH 8.0) until use.

### Enzyme assay

The catalytic activities of the purified wild-type and mutant xylanases were determined by measuring the amount of reducing sugar released from beechwood xylan by the 3,5-dinitrosalicylic acid (DNS) method, as described previously [32]. One unit (U) of xylanase activity was defined as the amount of enzyme required to release 1 μmol reducing sugar from xylan per min under the assay conditions. The pH profiles of the wild-type and mutants were determined at 55°C in different buffers from pH 4.5 to 10.0. The buffers used were citrate/Na_2_HPO_4_ buffer for pH 4.5–8.0, 0.05 M boric acid/borate for pH 8.0–9.0, and 0.05 M borate/NaOH for pH 9.5–10.0. All assays were performed in triplicate.

## Results

To understand the molecular basis for higher pH catalytic adaptation of family 11 xylanases, all selected xylanases with known pH-dependent activity (as reported in the literature) were mesophilic (optimum temperature between 40 and 60°C), to avoid confusing features of pH adaptation and temperature adaptation (S1 Table).

### Phylogenetic analysis of family 11 xylanases showed that bacterial xylanases have higher pH catalytic adaptation

A molecular phylogenetic tree of 68 xylanases is shown in S1 Fig. Major branches form two clusters, A and B. Cluster B is divided into clusters B1 and B2. Cluster A mainly consists of bacterial xylanases; cluster B1 comprises xylanases from bacteria (including actinomycetes) and fungi; cluster B2 comprises xylanases from fungi. From data on the characterization of xylanases, the pH optima of xylanases in cluster A is higher than of the enzymes in cluster B (S2 Table), and the pH optima of cluster B1 is higher than that of cluster B2 (S3 Table). Therefore, xylanases in clusters A, B1, and B2 are identified as alkaline, neutral, and acidic active xylanases, respectively. To study the molecular basis of higher pH catalytic adaptation of the xylanases, we compared the amino acid sequences, secondary structures, and tertiary structures of alkaline, neutral, and acidic active xylanases.

### Analysis of amino acid composition of family 11 xylanases indicates that a higher frequency of charged residues contributes to higher pH catalytic adaptation

Alteration of amino acid composition is related to protein adaptation to extreme environments [19]. Based on a comparison of the amino acid compositions between alkaline and non-alkaline xylanases, the alkaline active xylanases have more charged residues (Lys, Asp, and Glu) and a low ratio of negatively to positively charged residues (S2 Table). The higher frequency of charged residues may increase the number of polar interactions. An increase in hydrophobic residue content (Leu and Ile), and a decrease in polar residue (Ser, Thr, and Tyr) and Val content, are also observed in the alkaline active xylanases. These findings indicate that increasing the number of hydrophobic residues with large side chains and of charged residues, as well as decreasing the number of polar residues and hydrophobic residues with small side chains, may contribute to higher pH catalytic adaptation of family 11 xylanases.

On comparison of the amino acid compositions between neutral and acidic active xylanases, the most significant difference is in charged residue content (S3 Table). The acidophilic xylanases have fewer positively charged residues, which leads to a high ratio of negatively to positively charged residues. An increase in hydrophobic residue content is also observed in the acidophilic xylanases.

### Secondary structure comparison of family 11 xylanases elucidated that different sub-structures contribute significantly to higher pH catalytic adaptation

**Fig 1:**
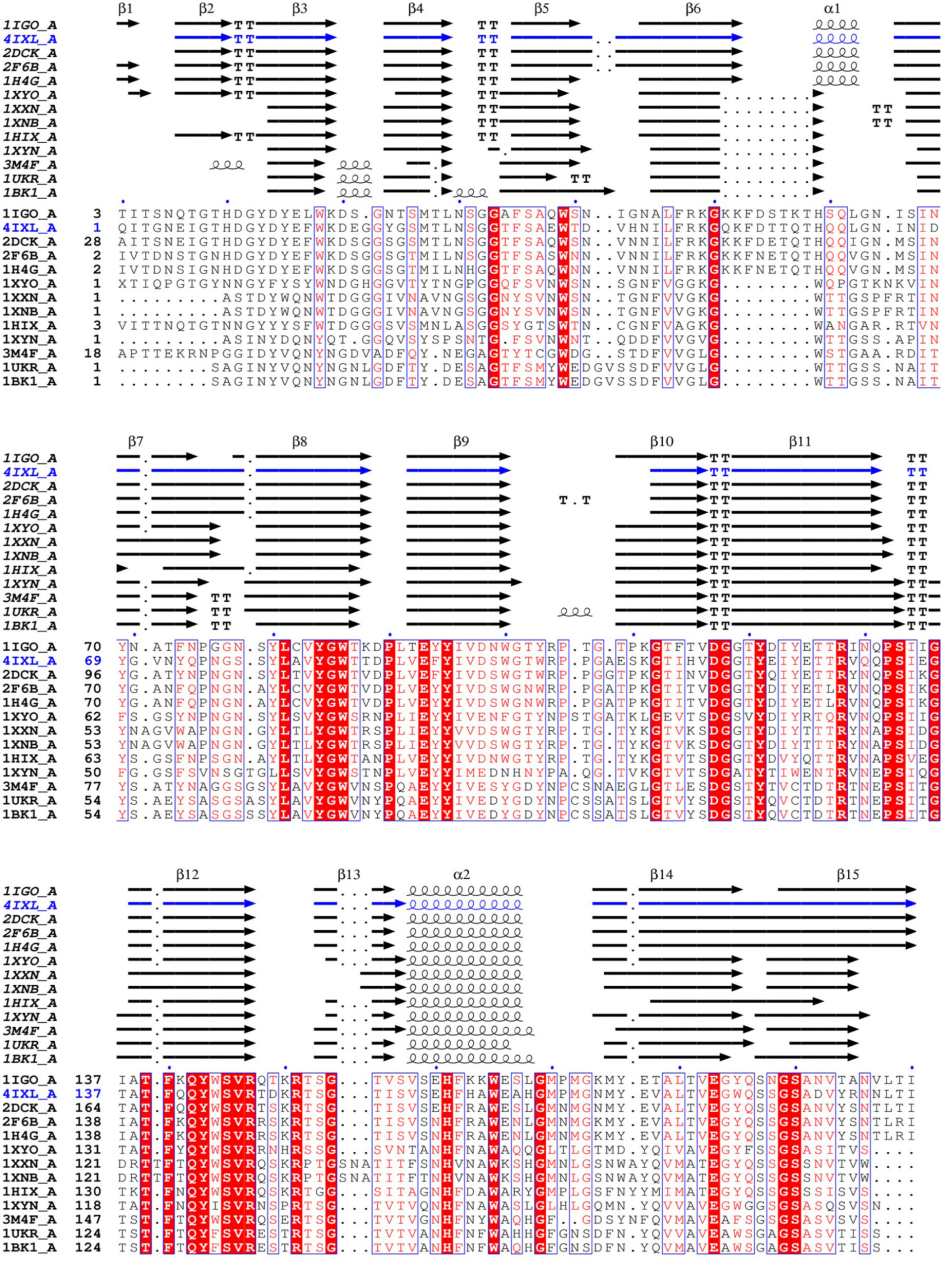
Structure-based sequence alignment of family 11 mesophilic xylanases with known structure and pH-dependent activity. Coils, arrows, and the symbol T represent helices, strands, and turns, respectively.

**Fig 2:**
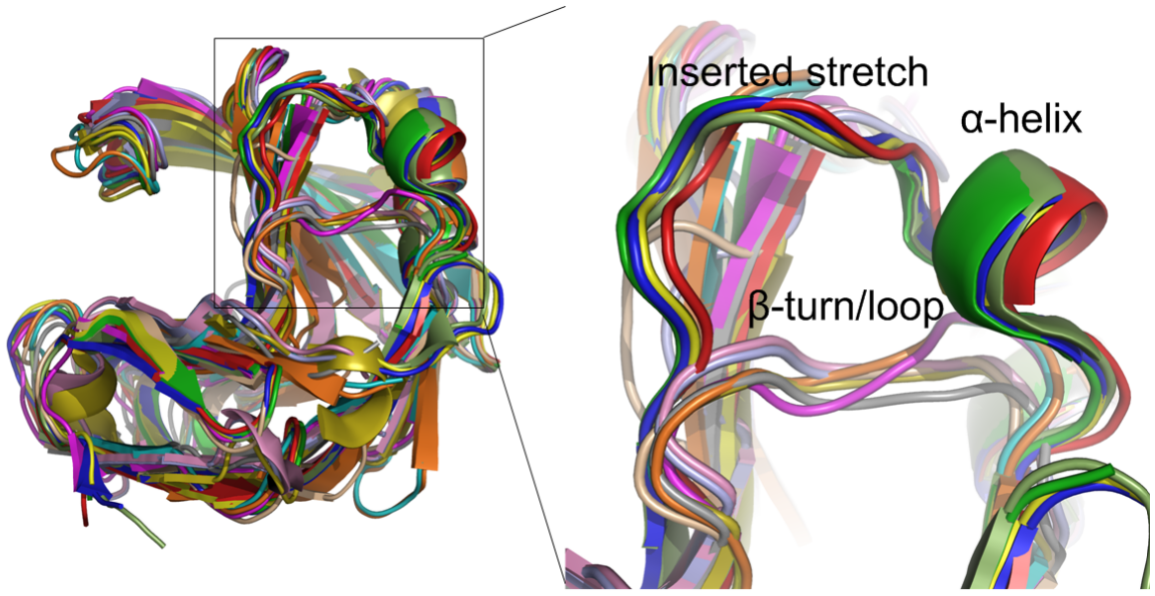
Three-dimensional structure superposition of family 11 mesophilic xylanases with known structure and pH-dependent activity. The difference in secondary structure between alkaline and non-alkaline xylanases is shown enlarged. PDB codes: 1IGO (pale green), 4IXL (red), 2DCK (green), 2F6B (blue), 1H4G (yellow), 1XYO (magenta), 1XXN (cyan), 1XNB (orange), 1HIX (wheat), 1XYN (gray), 3M4F (olive), 1UKR (light blue), and 1BK1 (pink).

To understand the structural basis of higher pH catalytic adaptation of family 11 xylanases, the three-dimensional structures of 13 family 11 mesophilic xylanases were obtained from the PDB. Based on phylogenetic analysis (see above), the 13 xylanases were divided into three groups, alkaline active xylanases, neutral active xylanases, and acidic active xylanases (Table 1). The secondary structure compositions of the three groups of enzymes are generally similar and mainly consist of β-sheet strands (Fig 1). However, there is a remarkable difference in secondary structures between the alkaline and non-alkaline xylanases. There is a helix α1 connecting strands β6 and β7 in the alkaline xylanases, while there is a β-turn or loop in the corresponding position in the non-alkaline xylanases (Fig 2). Alkaline xylanases have more hydrogen bonds in the α-helix between strands β6 and β7 than their non-alkaline counterparts have in the corresponding β-turn or loop (Table 1).

**Table 1:** The type of secondary structure and the number of hydrogen bonds between β6 and β7 of family 11 mesophilic xylanases with known structure and pH-dependent activity.

Enzyme abbreviation	Source	PDB code	Optimum pH	Phylogenetic analysis	The secondary structure between β6 and β7	The number of hydrogen bond
XynJ (ΔXBD)	*Bacillus* sp. 41M-1	2DCK	9[34]	Cluster A	α-helix	9
Xyl C	*alkalophilic Bacillus* (NCL 87-6-10)	2F6B	8[47]	Cluster A	α-helix	9
Xyn11X	*Bacillus subtilis* B230	1IGO	8[48]	Cluster A	α-helix	7
BadX	*Bacillus agaradhaerens* AC13	1H4G	8[49]	Cluster A	α-helix	9
Xyn11A-LC	*Bacillus* sp SN5	4IXL	7.5–8[21]	Cluster A	α-helix	8
XynA	*Bacillus subtilis* 168(1A1)	1XXN	6–6.5[50]	Cluster B1	β-turn	5
Xyl1	*Streptomyces* sp. S38	1HIX	6[51]	Cluster B1	loop	5
Bcx	*Bacillus circulans*	1XNB	5.7[52]	Cluster B1	β-turn	6
Xyn2	*Hypocrea jecorina* RUT-C30	1XYO	5.3[53]	Cluster B1	loop	10
XYNI	*Trichoderma reesei*	1XYN	3.5[42]	Cluster B2	loop	3
XYL1	*Scytalidium acidophilum*	3M4F	3.2[54]	Cluster B2	loop	7
XynA	*Aspergillus niger* CBS 513.88	1UKR	3[8]	Cluster B2	loop	2
XynC	*Aspergillus kawachii*	1BK1	2[55]	Cluster B2	loop	5

t = 4.667,P = 0.001.

**Fig 3:**
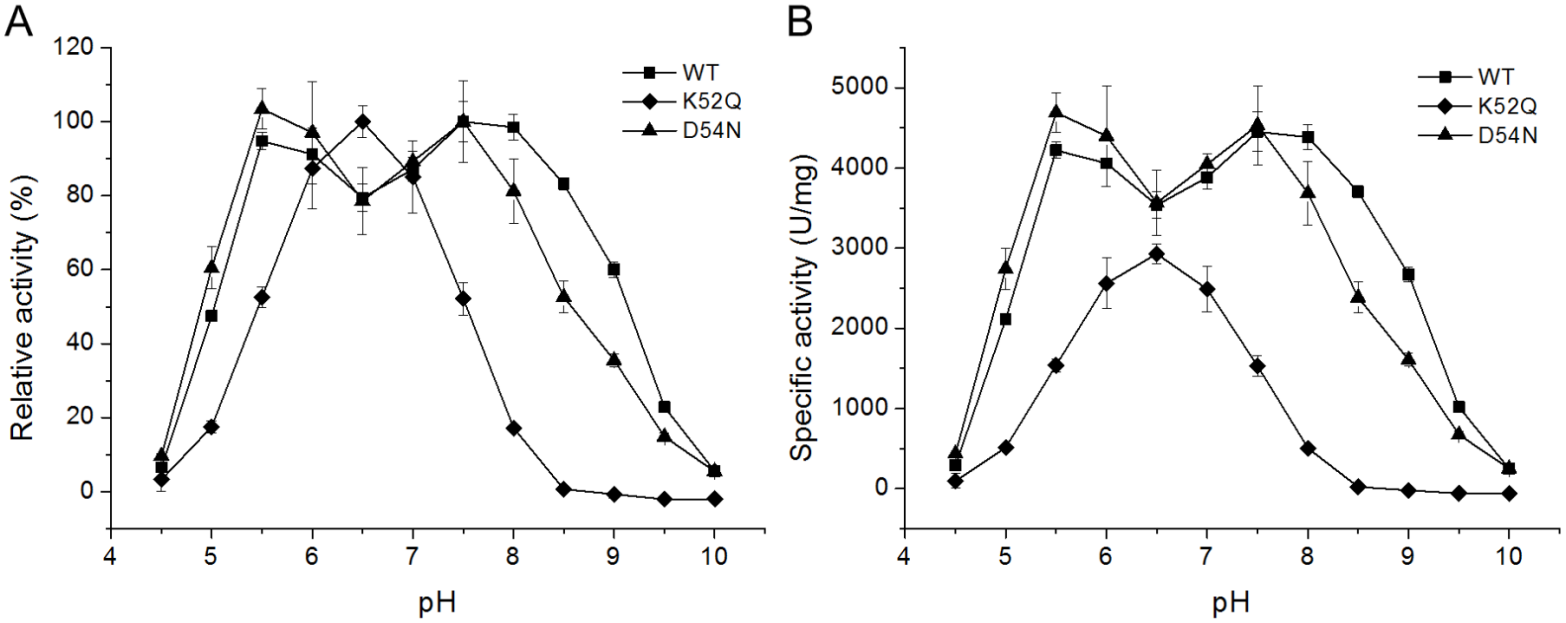
Effect of pH on the activity of wild-type Xyn11A-LC and the mutants K52Q and D54N. (A) pH-dependent relative activities of the wild type and the mutants. (B) pH-dependent specific activities of the wild type and the mutants.

In addition, the alkaline xylanases have an inserted stretch of seven amino acids rich in charged residues between strand β6 and helix α1 (Fig 1). To confirm the effect of these charged residues on alkaline adaptation of xylanases, the mutants K52Q and D54N of alkaline Xyn11A-LC were constructed. Recombinant Xyn11A-LC and the mutants were purified from crude extracts to electrophoretic homogeneity by Ni-affinity chromatography (S2 Fig). The pH-dependent activity profiles of the wild type and mutants are shown in Fig 3A. The mutation K52Q lowered the pH optimum from 7.5 to 6.5 and activity was nearly undetectable at pH 8.5. The pH profile of D54N was similar to that of the wild type at acidic and neutral pH, but its activity at alkaline pH (from 8.0 to 10.0) was lower than that of the wild type. The specific activity of the wild type was 4452 ± 247 U/mg at pH 7.5. The mutant K52Q exhibited decreased specific activity (2930 ± 92 U/mg) at its optimum pH (6.5), while the specific activity of D54N was 4532 ± 137 U/mg at pH 7.5, similar to that of the wild-type enzyme (Fig 3B). These mutational results preliminarily showed that the charged residues in the inserted stretch might contribute to the alkaline adaptation of family 11 xylanases. To confirm the role of helix α1 and the inserted stretch, we will introduce other mutations, such as truncation of the inserted stretch and replacement of the fragment between strands β6 and β7, in future work.

### Tertiary structure comparison of family 11 xylanases elucidated that solvent exposed amino acids and the number of ionic bonds affect higher pH catalytic adaptation

Proteins can adapt to extreme conditions if the amino acid content on the solvent-accessible surface is changed [19]. In family 11 xylanases, fewer acidic residues (Asp and Glu) and more basic residues (Lys and Arg) are on the surface of the alkaline and neutral enzymes except 4IXL from *Bacillus* sp. SN5, while more acidic residues and fewer basic residues are on the surface of acidophilic xylanases except 1XYN from *Trichoderma reesei* (Table 2). The Ser residue content, which is easily decomposed, is lower in the alkaline xylanases than in the non-alkaline enzymes. Acidophilic xylanases have more polar uncharged residues and fewer hydrophobic residues on their surfaces.

**Table 2:** Solvent-exposed residues, hydrogen bonds content and the number of ionic bonds of structure-determined family 11 mesophilic xylanases.

PDB code	Optimum pH	Acidic residues (%)	Basic residues (%)	Ser residue (%)	Uncharged, polar residues (%)	Hydrophobic residues (%)	Hydrogen bonds (%)	No. of ionic bonds
2DCK	9	7.3	9.8	9.8	58.5	24.4	70.3	6
2F6B	8	6.4	12.8	6.4	55.3	25.5	75.0	9
1IGO	8	9.1	11.4	9.1	56.8	22.7	78.3	9
1H4G	8	7.1	11.9	2.4	54.8	26.2	75.7	10
4IXL	7.5–8	23.8	4.8	2.4	45.2	26.2	69.7	6
1XXN	6–6.5	10.3	10.3	15.4	46.2	30.8	77.8	3
1HIX	6	2.3	9.1	9.1	65.9	22.7	70.3	3
1XNB	5.6	9.1	11.4	15.9	52.3	25.0	75.1	3
1XYO	5.3	2.5	7.5	2.5	55.0	32.5	73.7	4
1XYN	3.5	0	2.5	15.0	75.0	22.5	75.3	3
3M4F	3.2	29.0	0	9.7	58.1	12.9	79.3	1
1UKR	3	17.6	0	23.5	64.7	17.6	74.9	3
1BK1	2	18.6	0	23.3	60.5	20.9	74.9	2
mean		10.6	7.0	11.4	57.9	23.8	74.6	4.8

As shown in Table 2, no obvious difference in hydrogen bond content is observed among the alkaline, neutral, and acidic active xylanases, which indicates that there is no correlation between hydrogen bond content and pH-dependent activity of family 11 xylanases. However, the number of ionic bonds in the alkaline active xylanases is higher than that in non-alkaline active xylanases, indicating ionic bonds may be involved in alkaline adaptation of family 11 xylanases.

### Some important active site residues are key to higher pH catalytic adaptation

**Fig 4:**
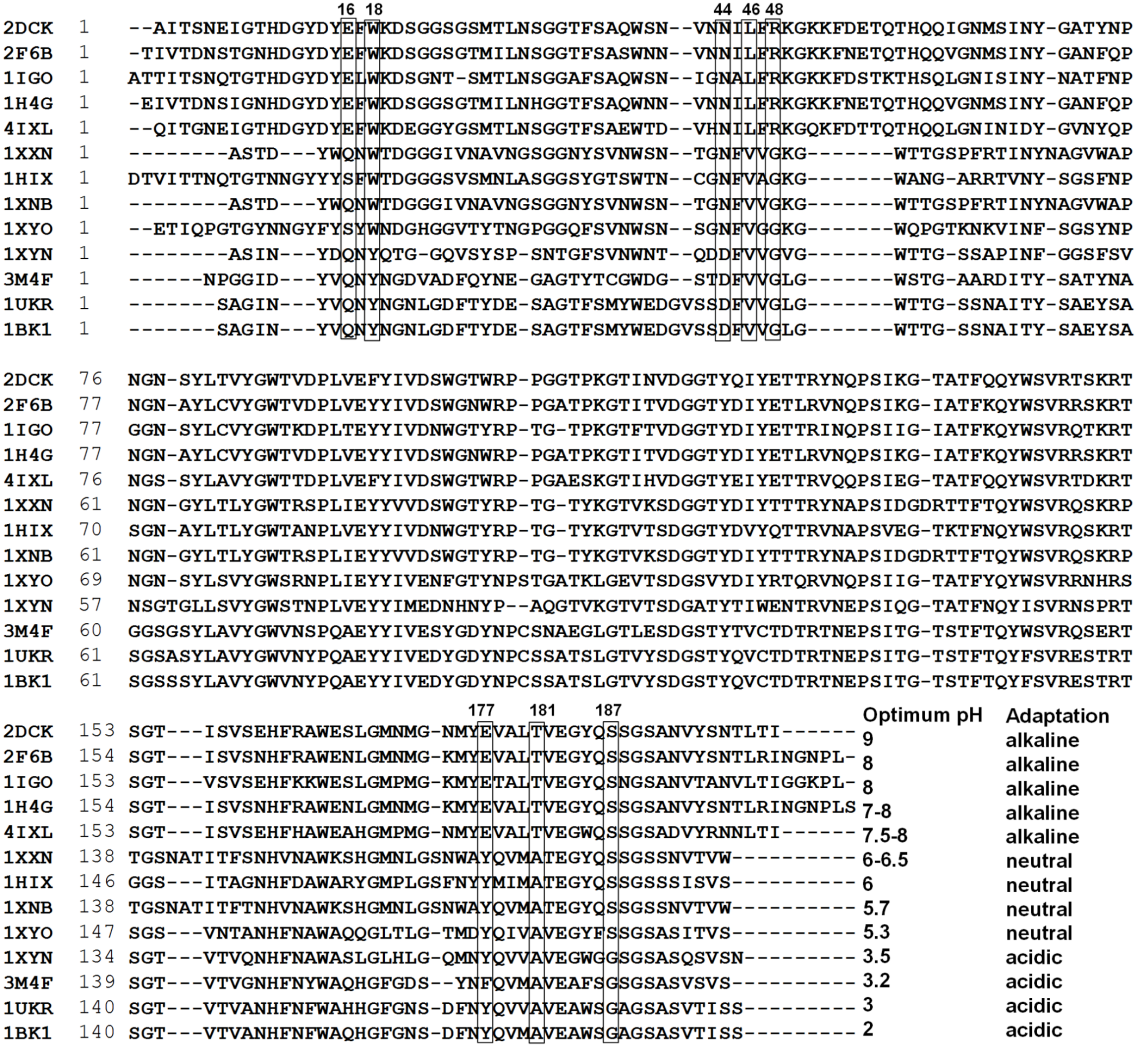
Sequence alignment of family 11 mesophilic xylanases with known structure and pH-dependent activity. The boxed amino acids indicate the key residues to determine the pH activity profile.

Amino acid residues within a radius of 12 Å from either of the two catalytic amino acids were identified as residues that may play important roles in determining the optimal pHs of the homologous family 11 xylanases. Thirty-one residues adjacent to the two catalytic amino acids are not conserved among the alkaline xylanase 2DCK, the neutral xylanase 1XXN, and the acidophilic xylanase 1BK1. Sequence alignment of thirteen xylanases with different optimal pH and known structure shows that eight conserved residues within the alkaline group may be the key amino acids determining the pH optima of family 11 xylanases (Fig 4).

**Fig 5:**
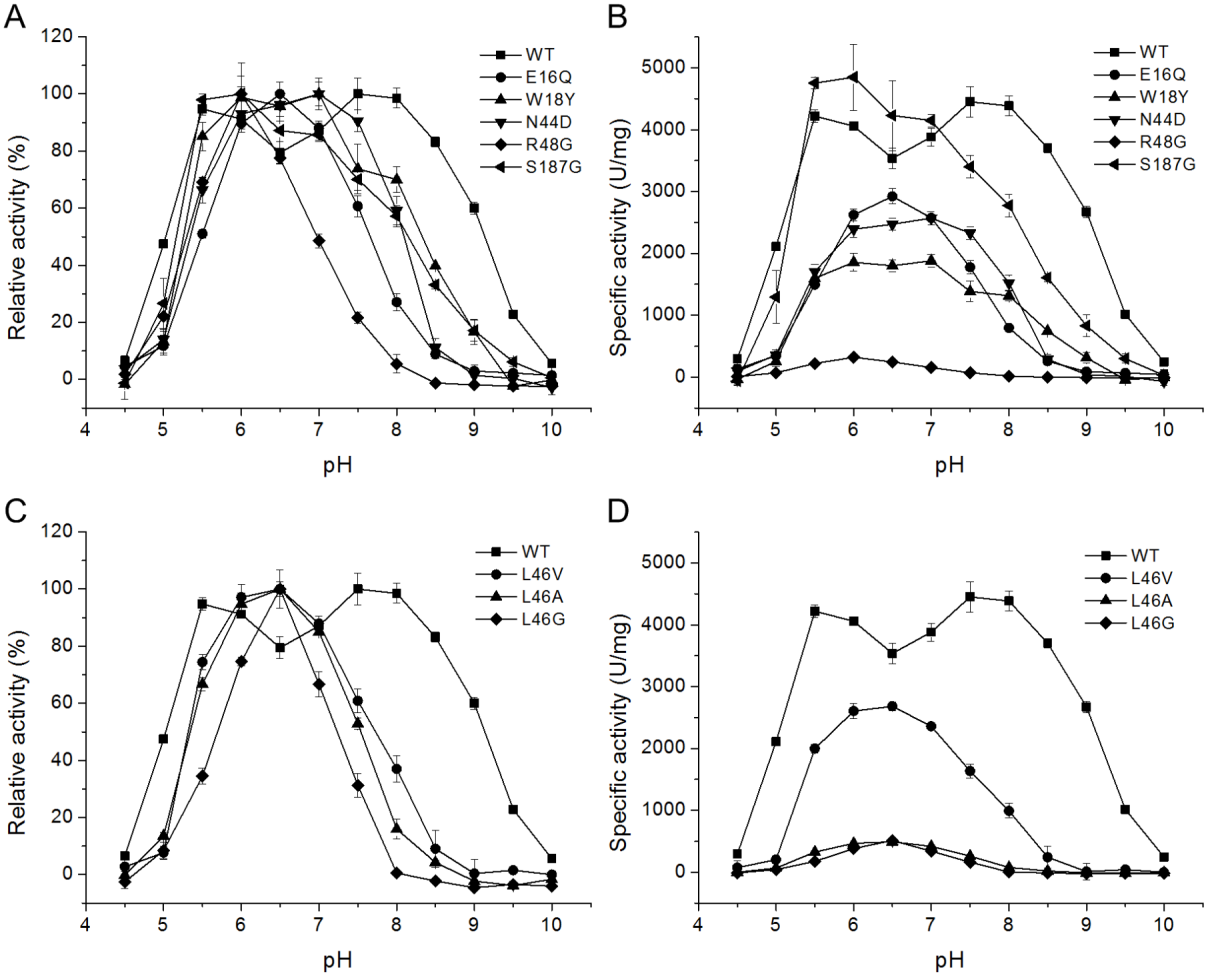
Effect of pH on the activity of wild-type Xyn11A-LC and mutants. (A) pH-dependent relative activities of the wild type and mutants E16Q, W18Y, N44D, R48G, and S187G. (B) pH-dependent specific activities of the wild type and mutants E16Q, W18Y, N44D, R48G, and S187G. (C) pH-dependent relative activities of the wild type and mutants L46V/A/G. (D) pH-dependent specific activities of the wild type and mutants L46V/A/G.

To evaluate the effect of these eight residues on the pH activity profiles of xylanases, mutations (E16Q, W18Y, N44D, L46V, R48G, E177Y, T181A, and S187G) of Xyn11A-LC were performed by site-directed mutagenesis and the mutants were characterized. The wild-type and mutants were purified from the crude extracts to electrophoretic homogeneity by Ni-affinity chromatography (S2 Fig). Fig 5 shows the pH activity profiles of the wild type and mutants E16Q, W18Y, N44D, L46V, R48G, and S187G. The optimum pH for catalysis by the wild-type enzyme was 7.5–8.0, and 60% residual activity was retained at pH 9.0. The pH activity profiles of the mutants E16Q, W18Y, N44D, L46V, R48G, and S187G were all shifted towards acidic pH values (Fig 5A and 5C). W18Y and N44D were maximally active at pH 7.0. W18Y retained 17% of its maximum activity at pH 9.0, but the activity of N44D was nearly undetectable at the same condition. The pH optima of E16Q and R48G decreased to 6.5 and 6.0, respectively, and their activities were negligible at pH 9.0 and pH 8.0, respectively. The optimum pH of S187G decreased to 5.5–6.0. The L46V mutant was optimally active at pH 6.5 and no activity could be detected at pH 9.0. In addition, Leu46 was mutated to other residues with smaller side chains such as Ala and Gly; the pH activity profile of L46A was similar to that of L46V, but L46G showed a narrower pH profile. The E177Y mutant was inactivate at all pHs tested, and the pH activity profile of T181A was similar to that of the wild-type enzyme. These results show that six amino acids in Xyn11A-LC, E16, W18, N44, L46, R48, and S187 might play an important role in catalysis by xylanase at higher pH.

All mutants except S187G exhibited decreased specific activities to varying degrees, relative to the wild-type enzyme, in their respective optimum pH conditions (Fig 5B and 5D). The specific activities of R48G, L46A, and L46G were 7.1%, 11.0%, and 11.4% of that of the wild-type enzyme, respectively.

## Discussion

### The relationship between amino acid composition and higher pH catalytic adaptation in family 11 xylanases

It is difficult to analyze the effect of each amino acid residue on the properties of the overall protein; nonetheless comparison of homologous proteins might reveal some trends in amino acid frequencies correlated to protein adaptation to extreme environments. Several studies have reported on the relationship of amino acid content to pH adaptation of enzymes [4,5,17–20]. For alkaline protease and cellulase, a decrease in the negatively charged residue (Asp and Glu) and Lys residue content, and an increase in Arg and neutral hydrophilic amino acid (His, Asn, and Gln) content, was observed during the course of alkaline adaptation [18,20]. An increase of Arg residue content was also observed in alkaline adaptation of the family 5 alkaline mannanase [19]. In contrast, family 10 alkaline active xylanase had an increase in negatively charged residue (Asp and Glu) content relative to non-alkaline enzyme, which resulted in a high ratio of negatively to positively charged residues [17]. Consistent with these observations on family 10 xylanases, family 11 alkaline xylanases also have increased negatively charged residue content relative to non-alkaline enzyme, but the latter also have an increase in Lys content, which results in a low ratio of negatively to positively charged residues. These results suggest that the mechanism of alkaline adaptation of an enzyme by changing the composition of amino acid residues might not be universal, but specific for each protein.

### The relationship between secondary structure and higher pH catalytic adaptation in family 11 xylanases

A helix α1 between strands β6 and β7 in the alkaline xylanases is substituted by a β-turn or loop in the non-alkaline counterparts (Fig 1). Further analysis indicates that the number of hydrogen bonds in this region of the alkaline xylanases is higher than that in their non-alkaline counterparts. We suggest that hydrogen bonds in this location might avoid instability introduced by the inserted stretch of seven amino acids between strands β6 and β7 in the alkaline xylanases.

Family 11 alkaline xylanases have an inserted stretch of seven amino acids rich in charged residues to adapt to alkaline conditions. In family 10 xylanases, the alkaline enzymes have three inserted stretches of >10 amino acids rich in negatively charged residues [4]. The alkaline mannanases have lost two inserted stretches of >10 amino acids rich in polar residues that were easily deaminated and oxidized at alkaline pH [19]. These observations suggest that alkaline adaptation of proteins can be acquired by inserting or deleting short sequences rich in particular types of residues, instead of gradual change over the entire protein chain [33]. In order to confirm the role of charged residues of the inserted stretch, the mutants K52Q and D54N of alkaline xylanase Xyn11A-LC were constructed. The mutants both lowered the catalytic activity under higher pH condition (Fig 3). Maybe the mutants changed the electrostatic and/or dynamic aspects of the active site, or their structure are unstable under alkaline condition. In order to verify the hypothesis, it will need a combination of kinetic, structural and ^13^C NMR studies. We will carry out detailed research on it in the future.

### The relationship between tertiary structure and higher pH catalytic adaptation in family 11 xylanases

Proteins can adapt to extreme conditions by changing amino acid content on the solvent-accessible surface [19]. The alteration of the solvent exposed residue content may be involved in adaptation to extreme pH. In family 11 xylanases, alkaline and neutral enzymes have more solvent-accessible positively charged residues (Lys and Arg), and fewer negatively charged residues (Asp and Glu), than acidophilic xylanases, in agreement with observations for M-protease and porcine pepsin [20]. Several mutagenesis studies also indicated that introduction of Arg residues into the Ser/Thr surface improved the alkalophilicity of xylanases [14,34]. The basic residues (Lys and Arg) have high p*K*_a_ values and can retain net positive charge even at high pH, which may result in high stability of enzymes with Lys/Arg rich surfaces at high pH. Nevertheless, alkaline active phosphoserine aminotransferase, mannanase, and family 10 xylanase have many negatively charged residues and a high ratio of negatively to positively charged residues on their surfaces [17,19,35]. These results suggest that changes in the ratio of negatively to positively charged residues on the surface may not be universal for alkaline adaptation of protein, but specific for different proteins.

An increase in the numbers of hydrogen bonds and ionic pairs can contribute to protein stability and may be involved in the adaptation of protein to extreme conditions. Alkalophilic M-proteases and aminotransferase have more hydrogen bonds than the non-alkalophilic enzymes. However, there is no correlation between hydrogen bond content and higher pH catalytic adaptation in family 11 xylanases. In agreement with our study, the hydrogen bond content of alkaline mannanases was similar to that of their non-alkaline counterparts [19]. These observations suggest that an increase in hydrogen bond content is favorable but not essential for high pH adaptation of proteins.

The correlation between the number of ionic bonds and extreme pH adaptation also varies. In this study, alkaline active xylanases have more ionic bonds than their non-alkaline active counterparts. Similar observations were made for the M-proteases and family 10 xylanases [17,20]. However, compared with their non-alkalophilic counterparts, the alkalophilic phosphoserine aminotransferases had fewer ionic bonds [35]. There was no obvious difference in the number of ionic bonds between alkalophilic and non-alkalophilic mannanases [19]. Further, alkaline adaptation of cellulase might not require an increase in the number of ionic bonds, but a remodeling of ion pairs from Lys-Asp to Arg-Asp [18]. This remodeling of ion pairs may be a general phenomenon in alkaline adaptation of protein, but an increase in the number of ion pairs is specific for each protein.

### The relationship between some important active site residues and higher pH catalytic adaptation in family 11 xylanases

In general, the pH activity profile of glycoside hydrolases is determined by the p*K*_*a*_ values of two catalytic residues: the nucleophile and the acid/base catalyst [36,37]. Some key amino acid residues in proximity to the catalytic residues can interact with them directly or indirectly and change their p*K*_*a*_ values by changing the electrostatic potential of the active site [36,38,39]. Consequently, these proximal amino acid residues are presumed to be responsible for the different pH activity profiles of homologous enzymes. In general, these key residues can be identified by structure comparison and sequence alignment of homologous proteins with different pH activity profiles. In the family 11 xylanases, eight residues within a radius of 12 Å of either of the two catalytic residues in Xyn11A-LC (Glu93 and Glu183) were selected and six (Glu16, Trp18, Asn44, Leu46, Arg48, and Ser187) were proved by mutational analysis to be key amino acids that determine the catalysis of Xyn11A-LC at higher pH.

**Fig 6:**
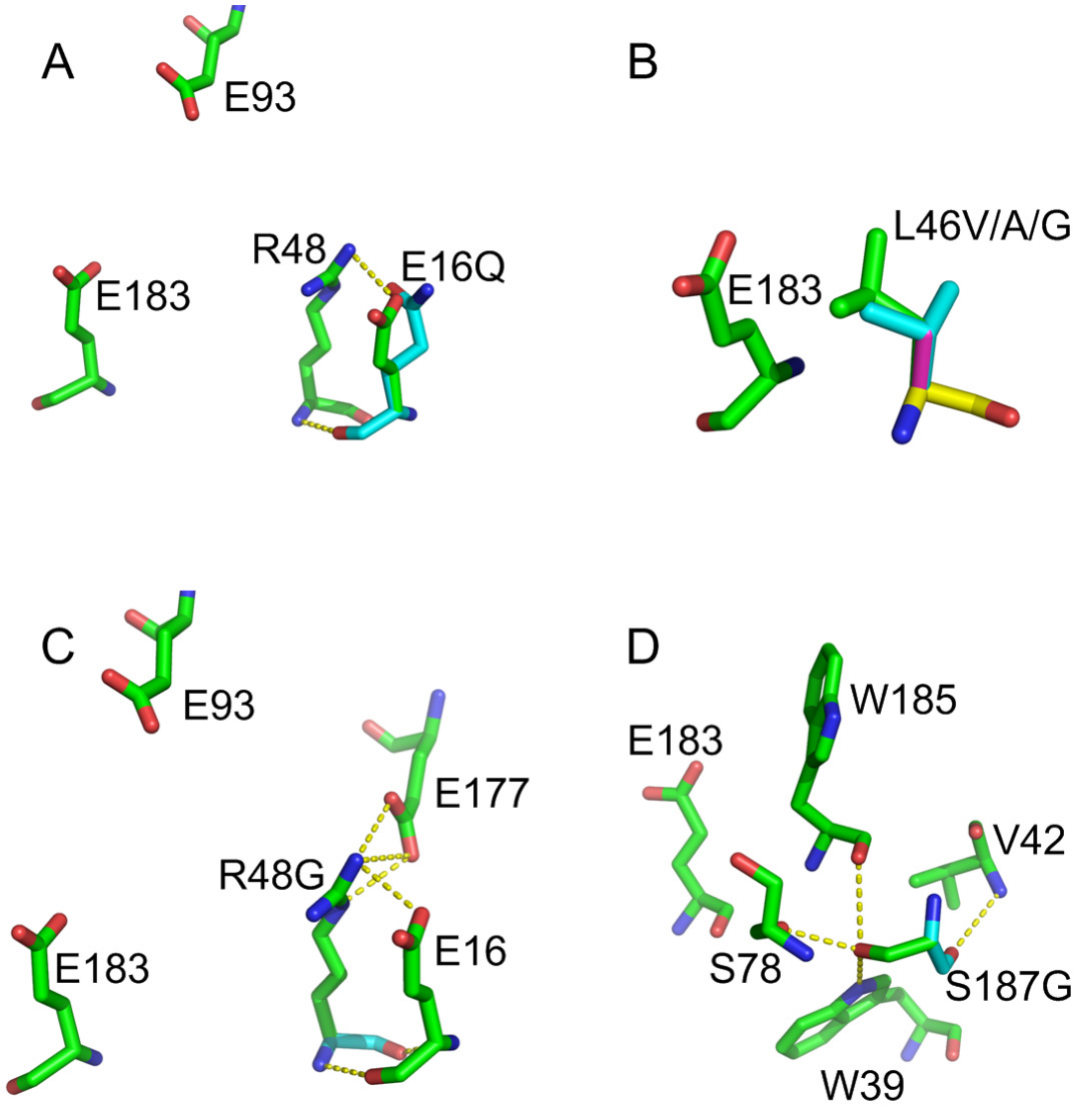
Structural models of six-point mutation sites around the catalytic center in Xyn11A-LC (PDB: 4IXL). (A) E16Q. (B) L46V/A/G. (C) R48G. (D) S187G. E16, L46, R48 and S187 are shown in green. The corresponding mutation sites are shown in cyan. V46, A46, and G46 are shown in cyan, magenta, and yellow, respectively. Hydrogen bonds and salt bridges are represented in yellow by dashed lines.

It is critical to maintain the protonation state of the acid/base catalyst when the enzymes function in alkaline conditions (i.e., low [H^+^]). In theory, amino acids with larger side chains could protect the positively charged group of the catalytic residues from the solvent [38]. The side chain of Leu46 is located 3.74 Å from the acid/base catalyst (Glu183); the loss of one methyl group in the mutant L46V might weaken the protective effect on the catalytic residue so that L46V had a lower pH optimum (Fig 6B). With a smaller side chain of residue 46, the enzyme was indeed more sensitive to the solvent. The mutant L46G had a narrower pH profile than L46V (Fig 5C). These results suggest that the side-chain group of residue 46 is responsible for the solvent accessibility of the acid/base catalyst. A decrease in the solvent accessibility of the acid/base catalyst may elevate its p*K*_*a*_ value by protecting it from hydroxide ions.

Local or overall negative charge can attract protons and raise the p*K*_*a*_ values of ionizable groups [40]. Therefore, the introduction of negatively charged residues (Asp and Glu) or the removal of positively charged residues (Arg and Lys) in the vicinity of the active site may increase the p*K*_*a*_ value of the catalytic residue. The mutation E16Q lowered the catalytic pH optimum relative to the wild type by decreasing the local negative charge and eliminating the salt bridge between Glu16 and Arg48 (Fig 6A). The mutation R48G removed a positively charged residue in proximity to the acid/base catalyst; this might be expected to increase the pH optimum to a more alkaline value. However, in mutational analysis of Xyn11A-LC, R48G variation decreased the pH optimum from 7.5 to 6.0. By structural analysis, it was found that the R48G mutation eliminated hydrogen bonds and ionic bonds between Arg48 and Glu16/Glu177 (Fig 6C). Therefore, the pH activity profile shift of the R48G mutant combines effects of charge and electrostatic interaction.

It was also counter-intuitive that the N44D mutation of Xyn11A-LC lowered the pH optimum from 7.5 to 7.0. However, several mutational studies of family 11 xylanases have confirmed this paradoxical result [41–45]. In the analysis of the high-resolution structure of BCX from *Bacillus circulans*, it was observed that the hydrogen bond distance (2.7 Å) between Asp35 O^δ2^ and the acid/base catalyst Glu172 O^ε2^ of the mutant N35D was shorter than that (3.3 Å) between Asn35 N^δ2^ and Glu172 O^ε2^ in the wild type. This shortening enhanced the hydrogen bond between Asp35 and Glu172, which tended to deprotonate the acid/base catalyst residue Glu172 and lowered its p*K*_*a*_ value [45]. Asn44 and Glu193 in Xyn11A-LC correspond to Asn35 and Glu172 in BCX from *B*. *circulans*, respectively.

The mutation W18Y decreased the alkalophilicity of Xyn11A-LC. A similar result was obtained for the xylanase Xyl1 from *Streptomyces* sp. S38, where mutation of W20Y of Xyl1 lowered the pH optimum of Xyl1 by one unit [46]. Modeling showed that the W20Y mutation led to the formation of a hydrogen bond between Tyr20 and Asn48, which induced a reduction of the distance between Asn48 N^δ2^ and the acid/base catalyst Glu191 O^ε1^, from 3.6 Å to 2.95 Å, and thereby decreased the p*K*_*a*_ value of Glu191 [46]. Trp18, Asn44, and Glu193 in Xyn11A-LC correspond to Trp20, Asn48, and Glu191 in Xyl1 from *Streptomyces* sp. S38, respectively.

The mutation S187G also decreased the alkalophilicity of Xyn11A-LC. The distance of Ser187 from the acid/base catalyst Glu183 is 9.35 Å. Ser187 can form hydrogen bonds with Trp39, Ser78, and Trp185 adjacent to Glu183 (Fig 6D). The mutation S187G might change the electrostatic potential of the active site by eliminating hydrogen bonds and thus lower the p*K*_*a*_ value of the catalytic residue.

From mutational analysis, we can draw the conclusion that the six amino acids Glu16, Trp18, Asn44, Leu46, Arg48, and Ser187 are important for catalysis by Xyn11A-LC at a higher pH. These amino acids might influence the electrostatic potential of the active site by changing the charge, hydrogen bonding, ionic bonding, or solvent accessibility around the catalytic residues and modify the p*K*_*a*_ values of the catalytic residues.

## Conclusions

By structural comparison and mutational analysis of xylanase Xyn11A-LC from alkalophilic *Bacillus* sp. SN5, the molecular mechanisms of higher pH catalytic adaptation of family 11 xylanases were studied systematically. Alkaline xylanases have increased charged residue content and decreased Ser, Thr, and Tyr residue content for activity at relatively high pHs. Between strands β6 and β7, different secondary structure elements may affect the pH-dependent activity of xylanases. One inserted stretch of seven amino acids rich in charged residues is probably beneficial for enzyme catalysis at relatively high pH. Positively charged residues are favored on the molecular surface of family 11 alkaline xylanases. Ionic bonds are beneficial for xylanases to adapt to an alkaline environment. Mutational analysis of Xyn11A-LC further revealed that at least six amino acids (E16, W18, N44, L46, R48, and S187) are involved in rendering the enzyme active at elevated pH. These findings help in understanding the molecular basis of higher pH catalytic adaptation of family 11 xylanases and may facilitate engineering of enzymes to suit industrial applications.

## Supporting Information

S1 Fig Phylogenetic tree of family 11 mesophilic xylanases with known pH-dependent activity. The numbers before the enzyme abbreviations indicate the pH optima. Alkaline active xylanases (Cluster A), neutral active xylanases (Cluster B1), and acidophilic xylanases (Cluster B2) are shown in red, blue, and black, respectively.
(TIF)

S2 Fig SDS-PAGE analysis of purified wild-type Xyn11A-LC and its variants. Lane 1, wild-type; lane 2, molecular weight markers; lanes 3–6, the mutants N44D, R48G, K52Q, and D54N, respectively.
(TIF)

S3 Fig Effect of pH on the stability of wild-type Xyn11A-LC and mutants. (TIF)

S1 Table All family 11 mesophilic xylanases with known pH-dependent activity. (DOC)

S2 Table Molecular feature comparison of family 11 xylanases grouped into different phylogenetic clusters (Clusters A and B). (DOC)

S3 Table Molecular feature comparison of family 11 xylanases grouped into different phylogenetic clusters (Clusters B1 and B2). (DOC)

## References

[1] Berrin JG, Juge N. Factors affecting xylanase functionality in the degradation of arabinoxylans. Biotechnology Letters. 2008;30(7):1139–50. 10.1007/s10529-008-9669-6 . 18320143

[2] Biely P. Microbial Xylanolytic Systems. Trends in biotechnology. 1985;3(11):286–90. 10.1016/0167-7799(85)90004-6 WOS:A1985AXN2600007.

[3] Henrissat B, G D. Structural and sequence based classification of glycosyl hydrolases. Current opinion in structural biology. 1997;7:637–44. 9345621 10.1016/s0959-440x(97)80072-3

[4] Manikandan K, Bhardwaj A, Gupta N, Lokanath NK, Ghosh A, Reddy VS, et al Crystal structures of native and xylosaccharide-bound alkali thermostable xylanase from an alkalophilic *Bacillus* sp. NG-27: structural insights into alkalophilicity and implications for adaptation to polyextreme conditions. Protein science: a publication of the Protein Society. 2006;15(8):1951–60. 10.1110/ps.062220206 16823036 PMC2242578

[5] Michaux C, Pouyez J, Mayard A, Vandurm P, Housen I, Wouters J. Structural insights into the acidophilic pH adaptation of a novel endo-1,4-beta-xylanase from *Scytalidium acidophilum* . Biochimie. 2010;92(10):1407–15. 10.1016/j.biochi.2010.07.003 WOS:000283637800018. 20621155

[6] Shallom D, Shoham Y. Microbial hemicellulases. Current opinion in microbiology. 2003;6(3):219–28. . 12831897 10.1016/s1369-5274(03)00056-0

[7] Beg QK, Kapoor M, Mahajan L, Hoondal GS. Microbial xylanases and their industrial applications: a review. Applied microbiology and biotechnology. 2001;56(3–4):326–38. WOS:000170608600004. 11548999 10.1007/s002530100704

[8] Krengel U, Dijkstra BW. Three-dimensional structure of endo-1,4-beta-xylanase I from *Aspergillus niger*: Molecular basis for its low pH optimum. Journal of molecular biology. 1996;263(1):70–8. 10.1006/jmbi.1996.0556 WOS:A1996VN40400006. 8890913

[9] Collins T, Gerday C, Feller G. Xylanases, xylanase families and extremophilic xylanases. Fems microbiology reviews. 2005;29(1):3–23. 10.1016/j.femsre.2004.06.005 WOS:000226703200002. 15652973

[10] Zhang S, Zhang K, Chen XZ, Chu X, Sun F, Dong ZY. Five mutations in N-terminus confer thermostability on mesophilic xylanase. Biochemical and biophysical research communications. 2010;395(2):200–6. 10.1016/j.bbrc.2010.03.159 WOS:000277681000008. 20361933

[11] Georis J, Esteves FD, Lamotte-Brasseur J, Bougnet V, Devreese B, Giannotta F, et al An additional aromatic interaction improves the thermostability and thermophilicity of a mesophilic family 11 xylanase: Structural basis and molecular study. Protein Science. 2000;9(3):466–75. WOS:000085994800004. 10752608 10.1110/ps.9.3.466 PMC2144569

[12] Xue H, Zhou J, You C, Huang Q, Lu H. Amino acid substitutions in the N-terminus, cord and alpha-helix domains improved the thermostability of a family 11 xylanase XynR8. Journal of industrial microbiology & biotechnology. 2012;39(9):1279–88. 10.1007/s10295-012-1140-y . 22584821

[13] Turunen O, Etuaho K, Fenel F, Vehmaanpera J, Wu XY, Rouvinen J, et al A combination of weakly stabilizing mutations with a disulfide bridge in the alpha-helix region of *Trichoderma reesei* endo-1,4-beta-xylanase II increases the thermal stability through synergism. Journal of biotechnology. 2001;88(1):37–46. 10.1016/S0168-1656(01)00253-X WOS:000169416200004. 11377763

[14] Turunen O, Vuorio M, Fenel F, Leisola M. Engineering of multiple arginines into the Ser/Thr surface of *Trichoderma reesei* endo-1,4-beta-xylanase II increases the thermotolerance and shifts the pH optimum towards alkaline pH. Protein engineering. 2002;15(2):141–5. 10.1093/protein/15.2.141 WOS:000174986500008. 11917150

[15] He J, Yu B, Zhang KY, Ding XM, Chen DW. Thermostable carbohydrate binding module increases the thermostability and substrate-binding capacity of *Trichoderma reesei* xylanase 2. New Biotechnology. 2009;26(1–2):53–9. 10.1016/j.nbt.2009.04.002 WOS:000271547200009. 19426845

[16] Hakulinen N, Turunen O, Janis J, Leisola M, Rouvinen J. Three-dimensional structures of thermophilic beta-1,4-xylanases from *Chaetomium thermophilum* and *Nonomuraea flexuosa*—Comparison of twelve xylanases in relation to their thermal stability. European journal of biochemistry. 2003;270(7):1399–412. 10.1046/j.1432-1033.2003.03496.x WOS:000181738200004. 12653995

[17] Mamo G, Thunnissen M, Hatti-Kaul R, Mattiasson B. An alkaline active xylanase: Insights into mechanisms of high pH catalytic adaptation. Biochimie. 2009;91(9):1187–96. 10.1016/j.biochi.2009.06.017 WOS:000269654100015. 19567261

[18] Shirai T, Ishida H, Noda J, Yamane T, Ozaki K, Hakamada Y, et al Crystal structure of alkaline cellulase K: insight into the alkaline adaptation of an industrial enzyme. Journal of molecular biology. 2001;310(5):1079–87. . 11501997 10.1006/jmbi.2001.4835

[19] Zhao Y, Zhang Y, Cao Y, Qi J, Mao L, Xue Y, et al Structural analysis of alkaline beta-mannanase from alkaliphilic *Bacillus* sp. N16-5: implications for adaptation to alkaline conditions. PloS one. 2011;6(1):e14608 10.1371/journal.pone.0014608 21436878 PMC3059134

[20] Shirai T, Suzuki A, Yamane T, Ashida T, Kobayashi T, Hitomi J, et al High-resolution crystal structure of M-protease: phylogeny aided analysis of the high-alkaline adaptation mechanism. Protein engineering. 1997;10(6):627–34. . 9278275 10.1093/protein/10.6.627

[21] Bai W, Xue Y, Zhou C, Ma Y. Cloning, expression, and characterization of a novel alkali-tolerant xylanase from alkaliphilic *Bacillus* sp. SN5. Biotechnology and applied biochemistry. 2015;62(2):208–17. 10.1002/bab.1265 . 24975401

[22] Bai W, Zhou C, Xue Y, Huang CH, Guo RT, Ma Y. Three-dimensional structure of an alkaline xylanase Xyn11A-LC from alkalophilic *Bacillus* sp. SN5 and improvement of its thermal performance by introducing arginines substitutions. Biotechnology letters. 2014;36(7):1495–501. 10.1007/s10529-014-1512-7 . 24682788

[23] Thompson JD, Higgins DG, Gibson TJ. Clustal-W—Improving the sensitivity of progressive multiple sequence alignment through sequence weighting, position-specific gap penalties and weight matrix choice. Nucleic acids research. 1994;22(22):4673–80. 10.1093/nar/22.22.4673 WOS:A1994PU19900018. 7984417 PMC308517

[24] Tamura K, Peterson D, Peterson N, Stecher G, Nei M, Kumar S. MEGA5: Molecular evolutionary genetics analysis using maximum likelihood, evolutionary distance, and maximum parsimony methods. Molecular biology and evolution. 2011;28(10):2731–9. 10.1093/molbev/msr121 WOS:000295184200003. 21546353 PMC3203626

[25] Saitou N, Nei M. The Neighbor-joining method—a new method for reconstructing phylogenetic trees. Molecular biology and evolution. 1987;4(4):406–25. WOS:A1987J406700007. 3447015 10.1093/oxfordjournals.molbev.a040454

[26] Rzhetsky A, Nei M. Theoretical foundation of the minimum-evolution method of phylogenetic inference. Molecular biology and evolution. 1993;10(5):1073–95. WOS:A1993LX26600011. 8412650 10.1093/oxfordjournals.molbev.a040056

[27] Guex N, Peitsch MC. SWISS-MODEL and the Swiss-PdbViewer: an environment for comparative protein modeling. Electrophoresis. 1997;18(15):2714–23. 10.1002/elps.1150181505 . 9504803

[28] Kabsch W, Sander C. Dictionary of protein secondary structure: pattern recognition of hydrogen-bonded and geometrical features. Biopolymers. 1983;22(12):2577–637. 10.1002/bip.360221211 . 6667333

[29] Robert X, Gouet P. Deciphering key features in protein structures with the new ENDscript server. Nucleic acids research. 2014;42(Web Server issue):W320–4. 10.1093/nar/gku316 24753421 PMC4086106

[30] Gouet P, Courcelle E, Stuart DI, Metoz F. ESPript: analysis of multiple sequence alignments in PostScript. Bioinformatics. 1999;15(4):305–8. . 10320398 10.1093/bioinformatics/15.4.305

[31] WL D. The PyMOL Molecular Graphics System. CA, USA: San Carlos: DeLano Scientific; 2002.

[32] Bai W, Xue Y, Zhou C, Ma Y. Cloning, expression and characterization of a novel salt-tolerant xylanase from *Bacillus* sp. SN5. Biotechnology letters. 2012;34(11):2093–9. 10.1007/s10529-012-1011-7 . 22864505

[33] Besir H, Zeth K, Bracher A, Heider U, Ishibashi M, Tokunaga M, et al Structure of a halophilic nucleoside diphosphate kinase from *Halobacterium salinarum* . Febs Letters. 2005;579(29):6595–600. 10.1016/j.febslet.2005.10.052 WOS:000233839500012. 16293253

[34] Umemoto H, Ihsanawati, Inami M, Yatsunami R, Fukui T, Kumasaka T, et al Improvement of alkaliphily of *Bacillus* alkaline xylanase by introducing amino acid substitutions both on catalytic cleft and protein surface. Bioscience biotechnology and biochemistry. 2009;73(4):965–7. 10.1271/Bbb.80869 WOS:000265739200035. 19352020

[35] Dubnovitsky AP, Kapetaniou EG, Papageorgiou AC. Enzyme adaptation to alkaline pH: Atomic resolution (1.08 A) structure of phosphoserine aminotransferase from *Bacillus alcalophilus* . Protein Science. 2005;14(1):97–110. 10.1110/Ps.041029805 WOS:000226384300011. 15608117 PMC2253317

[36] Joshi MD, Sidhu G, Nielsen JE, Brayer GD, Withers SG, McIntosh LP. Dissecting the electrostatic interactions and pH-dependent activity of a family 11 glycosidase. Biochemistry-Us. 2001;40(34):10115–39. 10.1021/Bi0105429 WOS:000170627800014. 11513590

[37] Nielsen JE, Mccammon JA. Calculating p*K* _*a*_ values in enzyme active sites. Protein Science. 2003;12(9):1894–901. 10.1110/Ps.03114903 WOS:000184976100009. 12930989 PMC2323987

[38] Shiraki K, Norioka S, Li SL, Yokota K, Sakiyama F. Electrostatic role of aromatic ring stacking in the pH-sensitive modulation of a chymotrypsin-type serine protease, *Achromobacter* protease I. European journal of biochemistry. 2002;269(16):4152–8. 10.1046/j.1432-1033.2002.03110.x WOS:000177782300030. 12180992

[39] Nielsen JE, Borchert TV, Vriend G. The determinants of alpha-amylase pH-activity profiles. Protein engineering. 2001;14(7):505–12. 10.1093/protein/14.7.505 WOS:000171021900009. 11522925

[40] Russell AJ, Fersht AR. Rational modification of enzyme catalysis by engineering surface-charge. Nature. 1987;328(6130):496–500. 10.1038/328496a0 WOS:A1987J481200049. 3302724

[41] Fushinobu S, Ito K, Konno M, Wakagi T, Matsuzawa H. Crystallographic and mutational analyses of an extremely acidophilic and acid-stable xylanase: biased distribution of acidic residues and importance of Asp37 for catalysis at low pH. Protein engineering. 1998;11(12):1121–8. 10.1093/protein/11.12.1121 WOS:000077987400001. 9930661

[42] Torronen A, Rouvinen J. Structural comparison of two major endo-1,4-xylanases from *Trichoderma reesei* . Biochemistry-Us. 1995;34(3):847–56. . 7827044 10.1021/bi00003a019

[43] Torronen A, Rouvinen J. Structural and functional properties of low molecular weight endo-1,4-beta-xylanases. Journal of biotechnology. 1997;57(1–3):137–49. . 9335170 10.1016/s0168-1656(97)00095-3

[44] Liu XM, Qu YB, You F, Liu Y. Studies on the key amino acid residues responsible for the alkali-tolerance of the xylanase by site-directed or random mutagenesis. Journal of molecular catalysis b-enzymatic. 2002;18(4–6):307–13. 10.1016/S1381-1177(02)00111-X WOS:000178982300016.

[45] Joshi MD, Sidhu G, Pot I, Brayer GD, Withers SG, McIntosh LP. Hydrogen bonding and catalysis: A novel explanation for how a single amino acid substitution can change the pH optimum of a glycosidase. Journal of molecular biology. 2000;299(1):255–79. 10.1006/jmbi.2000.3722 WOS:000087289400019. 10860737

[46] de Lemos Esteves F, Ruelle V, Lamotte-Brasseur J, Quinting B, Frere JM. Acidophilic adaptation of family 11 endo-beta-1,4-xylanases: modeling and mutational analysis. Protein science: a publication of the Protein Society. 2004;13(5):1209–18. 10.1110/ps.03556104 15096627 PMC2286771

[47] Balakrishnan H, Kamal Kumar B, Dutta-Choudhury M, Rele MV. Characterization of alkaline thermoactive cellulase-free xylanases from alkalophilic *Bacillus* (NCL 87-6-10). Journal of biochemistry, molecular biology, and biophysics: JBMBB: the official journal of the Federation of Asian and Oceanian Biochemists and Molecular Biologists. 2002;6(5):325–34. 10.1080/1025814021000003229 . 12385968

[48] Oakley AJ, Heinrich T, Thompson CA, Wilce MCJ. Characterization of a family 11 xylanase from *Bacillus subtillis* B230 used for paper bleaching. Acta crystallographica section d-biological crystallography. 2003;59:627–36. 10.1107/S0907444903001227 WOS:000181815600003. 12657781

[49] Poon DK, Webster P, Withers SG, McIntosh LP. Characterizing the pH-dependent stability and catalytic mechanism of the family 11 xylanase from the alkalophilic *Bacillus agaradhaerens* . Carbohydrate research. 2003;338(5):415–21. . 12559743 10.1016/s0008-6215(02)00486-x

[50] Ruller R, Rosa JC, Faca VM, Greene LJ, Ward RJ. Efficient constitutive expression of *Bacillus subtilis* xylanase A in *Escherichia coli* DH5α under the control of the *Bacillus* BsXA promoter. Biotechnology and applied biochemistry. 2006;43(Pt 1):9–15. 10.1042/BA20050016 . 15982188

[51] Georis J, Giannotta F, De Buyl E, Granier B, Frere JM. Purification and properties of three endo-beta-1,4-xylanases produced by *Streptomyces* sp strain S38 which differ in their ability to enhance the bleaching of kraft pulps. Enzyme and microbial technology. 2000;26(2–4):178–86. 10.1016/S0141-0229(99)00141-6 WOS:000085509700012. 10689075

[52] McIntosh LP, Hand G, Johnson PE, Joshi MD, Korner M, Plesniak LA, et al The p*K* _*a*_ of the general acid/base carboxyl group of a glycosidase cycles during catalysis: a ^13^C-NMR study of *Bacillus circulans* xylanase. Biochemistry-Us. 1996;35(31):9958–66. 10.1021/bi9613234 . 8756457

[53] Torronen A, Harkki A, Rouvinen J. Three-dimensional structure of endo-1,4-beta-xylanase II from *Trichoderma reesei*: two conformational states in the active site. The EMBO journal. 1994;13(11):2493–501. 8013449 10.1002/j.1460-2075.1994.tb06536.x PMC395120

[54] Al Balaa B, Wouters J, Dogne S, Rossini C, Schaus JM, Depiereux E, et al Identification, cloning, and expression of the *Scytalidium acidophilum* XYL1 gene encoding for an acidophilic xylanase. Bioscience, biotechnology, and biochemistry. 2006;70(1):269–72. 10.1271/bbb.70.269 . 16428847

[55] Ito K, Ogasawara H, Sugimoto T, Ishikawa T. Purification and properties of acid stable xylanases from *Aspergillus-Kawachii* . Bioscience biotechnology and biochemistry. 1992;56(4):547–50. WOS:A1992HT31000002. 10.1271/bbb.56.547 27280644

