# Supplementary figures and images for: Structural Insight into and Mutational Analysis of Family 11 Xylanases: Implications for Mechanisms of Higher pH Catalytic Adaptation
Source: PLoS One. 2015 Jul 10;10(7):e0132834. doi: 10.1371/journal.pone.0132834 (PMC4498622; doi:10.1371/journal.pone.0132834)

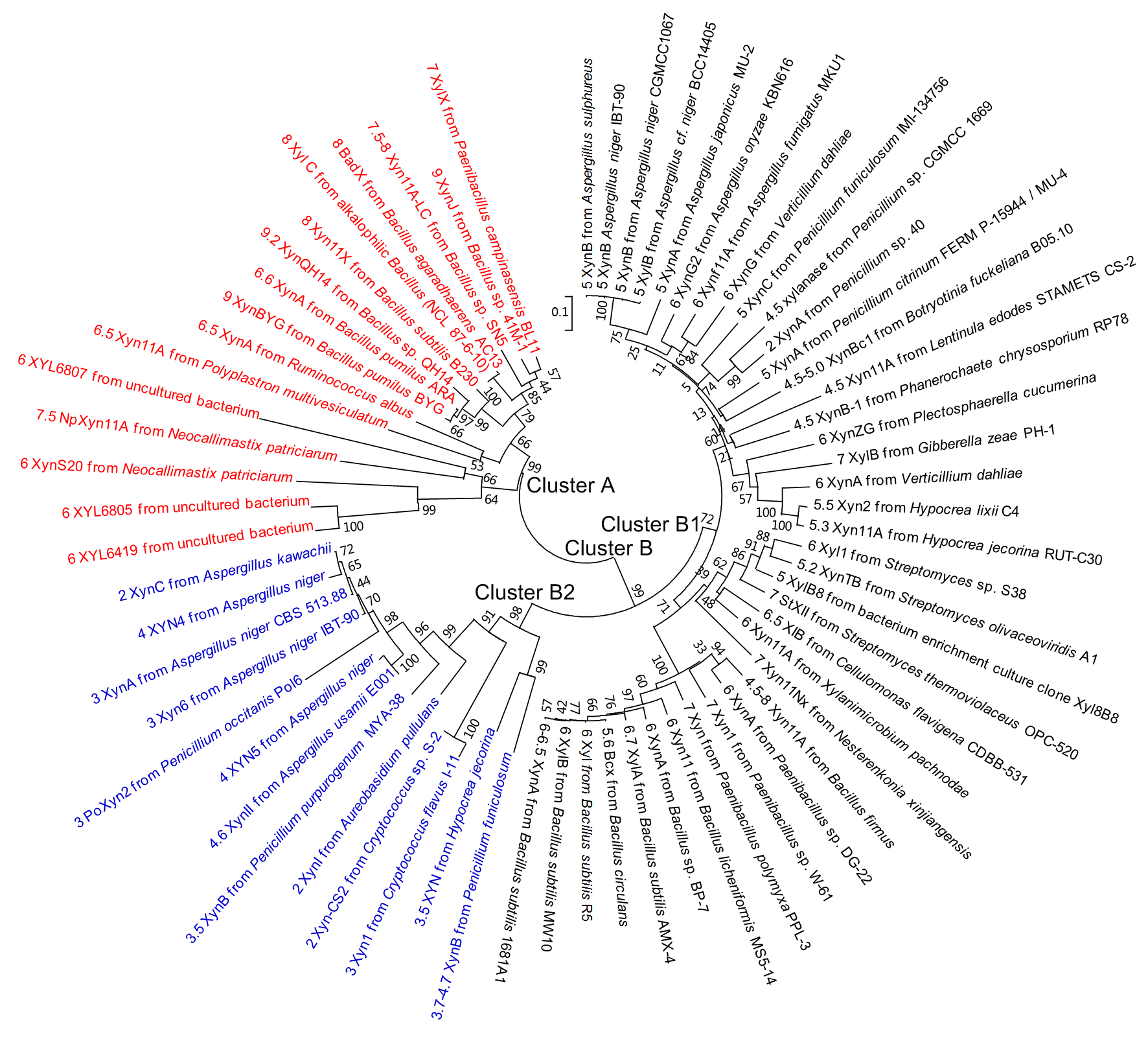

Supplement: S1 Fig — The numbers before the enzyme abbreviations indicate the pH optima. Alkaline active xylanases (Cluster A), neutral active xylanases (Cluster B1), and acidophilic xylanases (Cluster B2) are shown in red, blue, and black, respectively. (TIF) [file pone.0132834.s001.tif]

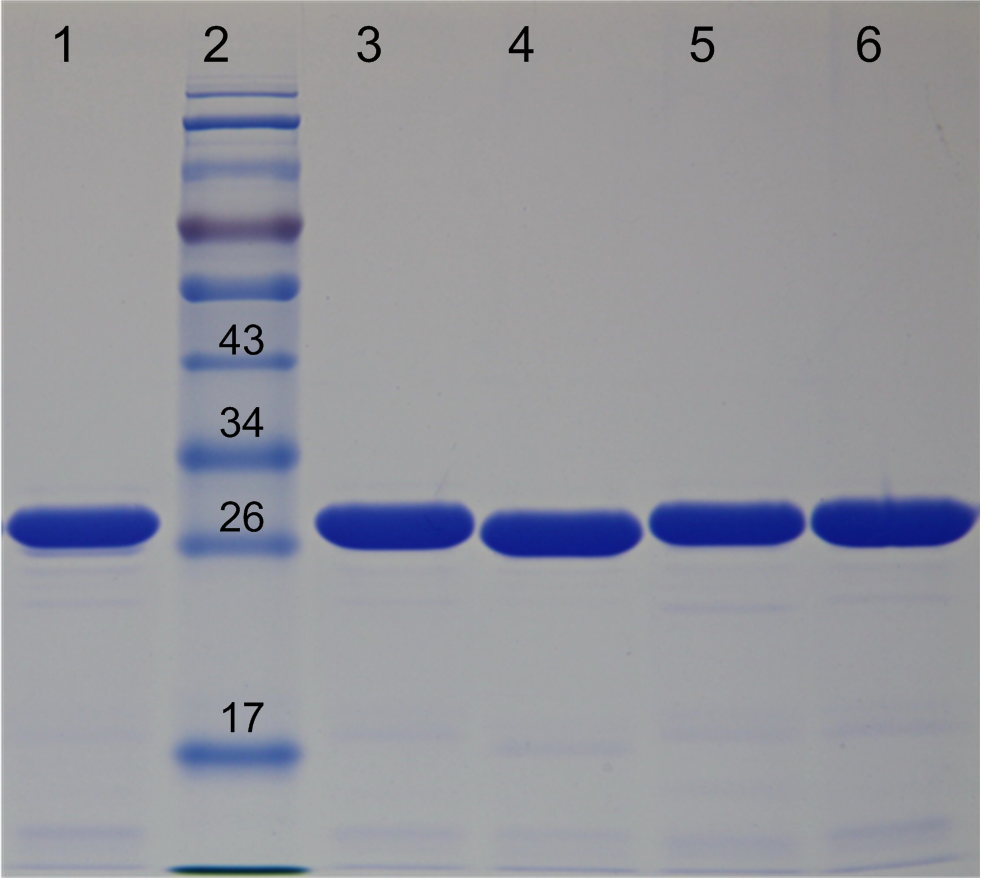

Supplement: S2 Fig — Lane 1, wild-type; lane 2, molecular weight markers; lanes 3–6, the mutants N44D, R48G, K52Q, and D54N, respectively. (TIF) [file pone.0132834.s002.tif]

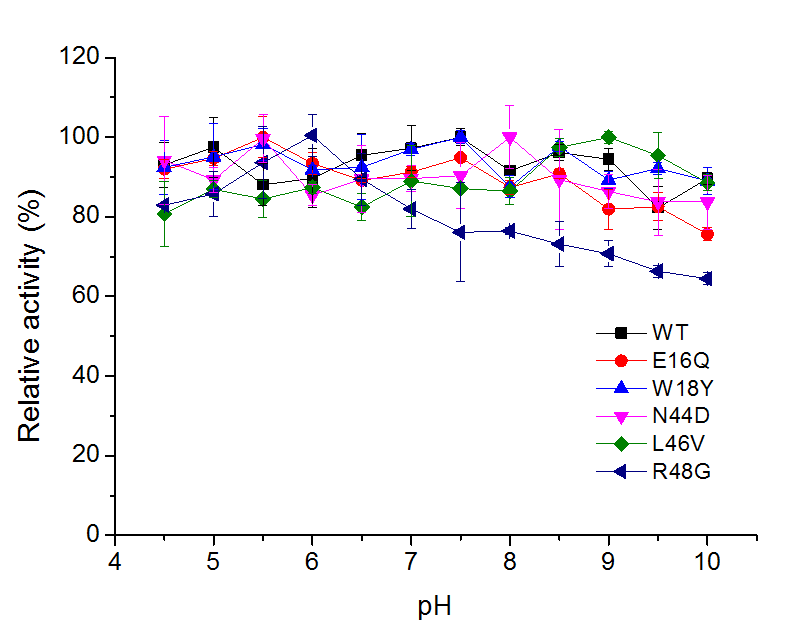

Supplement: S3 Fig — (TIF) [file pone.0132834.s003.tif]
