# Supplementary material for: Structural Insight into and Mutational Analysis of Family 11 Xylanases: Implications for Mechanisms of Higher pH Catalytic Adaptation
Source: PLoS One. 2015 Jul 10;10(7):e0132834. doi: 10.1371/journal.pone.0132834 (PMC4498622; doi:10.1371/journal.pone.0132834)
Supplement: S1 Table — (DOC) [file pone.0132834.s004.doc]

Table S1 all pH-dependent activity characterized and mesophilic GH 11 xylanases

| Enzyme abbreviation | Source | Optimum temperature(℃) | Optimum pH | Ref. |
| --- | --- | --- | --- | --- |
| XynQH14 | *Bacillus.*sp.QH14 | 60 | 9.2 |  |
| XynJ* | [*Bacillus* sp. 41M-1](http://www.ncbi.nlm.nih.gov/Taxonomy/Browser/wwwtax.cgi?id=98930) | 55 | 9 |  |
| XynBYG | *Bacillus pumilus* BYG | 50 | 9 |  |
| Xyl C* | alkalophilic *Bacillus* (NCL 87-6-10) | 40-60 | 8 |  |
| Xyn11X* | [*Bacillus subtilis* B230](http://www.ncbi.nlm.nih.gov/Taxonomy/Browser/wwwtax.cgi?id=1423) | 60 | 8 |  |
| BadX* | [*Bacillus agaradhaerens* AC13](http://www.ncbi.nlm.nih.gov/Taxonomy/Browser/wwwtax.cgi?id=76935) | 55 | 8 |  |
| Xyn11A-LC* | *Bacillus* sp. SN5 | 55 | 7.5-8 |  |
| Xyn11A | [*Bacillus firmus*](http://www.ncbi.nlm.nih.gov/Taxonomy/Browser/wwwtax.cgi?id=1399) | 60 | 4.5-8 |  |
| NpXyn11A | [*Neocallimastix patriciarum*](http://www.ncbi.nlm.nih.gov/Taxonomy/Browser/wwwtax.cgi?id=4758) | N/A | 7.5 |  |
| StXII | [*Streptomyces thermoviolaceus* OPC-520](http://www.ncbi.nlm.nih.gov/Taxonomy/Browser/wwwtax.cgi?id=1952) | 60 | 7 |  |
| XylX | [*Paenibacillus campinasensis* BL11](http://www.ncbi.nlm.nih.gov/Taxonomy/Browser/wwwtax.cgi?id=66347) | 60 | 7 |  |
| Xyn11Nx | [*Nesterenkonia xinjiangensis* YIM 70097 / CCTCC AA001025](http://www.ncbi.nlm.nih.gov/Taxonomy/Browser/wwwtax.cgi?id=225327) | 55 | 7 |  |
| Xyn1 | [*Paenibacillus* sp. W-61](http://www.ncbi.nlm.nih.gov/Taxonomy/Browser/wwwtax.cgi?id=231952) | 55 | 7 |  |
| Xyn | [*Paenibacillus polymyxa* PPL-3](http://www.ncbi.nlm.nih.gov/Taxonomy/Browser/wwwtax.cgi?id=1406) | 40 | 7 |  |
| XylB | *Gibberella zeae* PH-1 | 35 | 7 |  |
| XylA | [*Bacillus subtilis* AMX-4](http://www.ncbi.nlm.nih.gov/Taxonomy/Browser/wwwtax.cgi?id=1423) | 50-55 | 6-7 |  |
| XynA | [*Bacillus pumilus* ARA](http://www.ncbi.nlm.nih.gov/Taxonomy/Browser/wwwtax.cgi?id=1408) | 50 | 6.6 |  |
| XIB | [*Cellulomonas flavigena* CDBB-531](http://www.ncbi.nlm.nih.gov/Taxonomy/Browser/wwwtax.cgi?id=1711) | 55 | 6.5 |  |
| Xyn11A | [*Polyplastron multivesiculatum*](http://www.ncbi.nlm.nih.gov/Taxonomy/Browser/wwwtax.cgi?id=47894) | 40 | 6.5 |  |
| XynA | [*Ruminococcus albus*](http://www.ncbi.nlm.nih.gov/Taxonomy/Browser/wwwtax.cgi?id=1264) | N/A | 6.5 |  |
| XynA* | [*Bacillus subtilis* 168](http://www.cazy.org/b68.html)（1A1） | 55 | 6-6.5 |  |
| XylB | [*Bacillus subtilis* MW10](http://www.ncbi.nlm.nih.gov/Taxonomy/Browser/wwwtax.cgi?id=1423) | 60 | 6 |  |
| Xyn11 | *Bacillus licheniformis* | 40-50 | 6 |  |
| XynA | [*Bacillus* sp. BP-7](http://www.ncbi.nlm.nih.gov/Taxonomy/Browser/wwwtax.cgi?id=126733) | 60 | 6 |  |
| XynA | *Paenibacillus* sp. DG-22 | 60 | 6 |  |
| Xynf11A | *Aspergillus fumigatus* MKU1 | 60 | 6 |  |
| Xyl1* | *Streptomyces* sp. S38 | 60 | 6 |  |
| XynG2 | *Aspergillus oryzae* KBN616 | 58 | 6 |  |
| Xyl | *Bacillus subtilis* R5 | 40-50 | 6 |  |
| XYL6419 | uncultured bacterium | 50 | 6 |  |
| XYL6807 | uncultured bacterium | 50 | 6 |  |
| XYL6805 | uncultured bacterium | 50 | 6 |  |
| Xyn11A | *Xylanimicrobium pachnodae* | 50 | 6 |  |
| XynS20 | *Neocallimastix patriciarum* | 45 | 6 |  |
| XynG | *Verticillium dahliae* | 45 | 6 |  |
| XynA | *Trichoderma* sp. SC9 | 42.5 | 6 |  |
| XynZG | *Plectosphaerella cucumerina* | 40 | 6 |  |
| Bcx* | [*Bacillus circulans*](http://www.ncbi.nlm.nih.gov/Taxonomy/Browser/wwwtax.cgi?id=1397) | N/A | 5.7 |  |
| Xyn2 | *Hypocrea lixii* C4 | 50 | 5.5 | [37] |
| Xyn2* | [*Hypocrea jecorina* RUT-C30](http://www.ncbi.nlm.nih.gov/Taxonomy/Browser/wwwtax.cgi?id=51453) | N/A | 5.3 | [38] |
| XynTB | [*Streptomyces olivaceoviridis* A1](http://www.ncbi.nlm.nih.gov/Taxonomy/Browser/wwwtax.cgi?id=1921) | 60 | 5.2 | [39] |
| XynC | [*Penicillium funiculosum* IMI-134756](http://www.ncbi.nlm.nih.gov/Taxonomy/Browser/wwwtax.cgi?id=28572) | 55 | 5 |  |
| XylB8 | [bacterium enrichment culture clone Xyl8B8](http://www.ncbi.nlm.nih.gov/Taxonomy/Browser/wwwtax.cgi?id=1169993) | 55 | 5 |  |
| XylB | [*Aspergillus cf. niger* BCC14405](http://www.ncbi.nlm.nih.gov/Taxonomy/Browser/wwwtax.cgi?id=266074) | 55 | 5 |  |
| XynA | *Penicillium citrinum* FERM P-15944 / MU-4 | 55 | 5 |  |
| XynA | *Aspergillus japonicus* MU-2 | 60 | 5 |  |
| XynB | *Aspergillus niger* CGMCC1067 | 50 | 5 |  |
| XynB | *Aspergillus sulphureus* | 50 | 5 |  |
| XynB | *Aspergillus niger* IBT-90 | N/A | 5 | [47] |
| XynBc1 | *Botryotinia fuckeliana* B05.10 | 38-42 | 4.5-5.0 | [48] |
| XynII | *Aspergillus usamii* E001 | 50 | 4.6 | [49] |
| Xyn11A | *Lentinula edodes* STAMETS CS-2 | 50 | 4.5 |  |
| xylanase | *Penicillium* sp. CGMCC 1669 | 40 | 4.5 |  |
| XynB-1 | *Phanerochaete chrysosporium* RP78 | 60 | 4.5 |  |
| XynB | *Penicillium funiculosum* | N/A | 3.7-4.7 |  |
| XYN4 | *Aspergillus niger* | 60 | 4 |  |
| XYN5 | *Aspergillus niger* | 60 | 4 |  |
| XynB | *Penicillium purpurogenum* MYA-38 | 50 | 3.5 |  |
| XYNⅠ* | *Hypocrea jecorina* | N/A | 3.5 |  |
| XYL1* | *Scytalidium acidophilum* | 56 | 3.2 | [57] |
| Xyn6 | *Aspergillus niger* IBT-90 | N/A | 3 | [47] |
| Xyn1 | *Cryptococcus flavus* I-11 | 50 | 3 | [58] |
| Xyn2 | *Penicillium occitanis* Pol6 | 50 | 3 | [59] |
| XynA* | *Aspergillus niger* CBS 513.88 | N/A | 3 |  |
| XynI | *Aureobasidium pullulans var. melanogenum* | 50 | 2 |  |
| Xyn-CS2 | *Cryptococcus* sp. S-2 | 40 | 2 |  |
| XynA | *Penicillium* sp. 40 | 50 | 2 |  |
| XynC* | *Aspergillus kawachii* | 50 | 2 |  |

NA = not available. * 3D structure has been solved.

References

1. Shan Z, Zhou J, Zhou Y, Yuan H, Lv H (2012 ) Isolation and characterization of an alkaline xylanase from a newly isolated *Bacillus* sp. QH14. Yi Chuan 34: 356-365.

2. Umemoto H, Ihsanawati, Inami M, Yatsunami R, Fukui T, et al. (2009) Improvement of alkaliphily of *Bacillus* alkaline xylanase by introducing amino acid substitutions both on catalytic cleft and protein surface. Biosci Biotech Bioch 73: 965-967.

3. Wang J, Zhang WW, Liu JN, Cao YL, Bai XT, et al. (2010) An alkali-tolerant xylanase produced by the newly isolated alkaliphilic *Bacillus pumilus* from paper mill effluent. Mol Biol Rep 37: 3297-3302.

4. Balakrishnan H, Kamal Kumar B, Dutta-Choudhury M, Rele MV (2002) Characterization of alkaline thermoactive cellulase-free xylanases from alkalophilic *Bacillus* (NCL 87-6-10). J Biochem Mol Biol Biophys 6: 325-334.

5. Oakley AJ, Heinrich T, Thompson CA, Wilce MCJ (2003) Characterization of a family 11 xylanase from *Bacillus subtillis* B230 used for paper bleaching. Acta Crystallogr D 59: 627-636.

6. Poon DKY, Webster P, Withers SG, McIntosh LP (2003) Characterizing the pH-dependent stability and catalytic mechanism of the family 11 xylanase from the alkalophilic *Bacillus agaradhaerens*. Carbohydr Res 338: 415-421.

7. Bai W, Xue Y, Zhou C, Ma Y (2014) Cloning, expression, and characterization of a novel alkali-tolerant xylanase from alkaliphilic *Bacillus* sp. SN5. Biotechnol Appl Biochem. doi: 10.1002/bab.1265.

8. Chang PC, Tsai WS, Tsai CL, Tseng MJ (2004) Cloning and characterization of two thermostable xylanases from an alkaliphilic *Bacillus firmus*. Biochem Bioph Res Co 319: 1017-1025.

9. Vardakou M, Dumon C, Murray JW, Christakopoulos P, Weiner DP, et al. (2008) Understanding the structural basis for substrate and inhibitor recognition in eukaryotic GH11 xylanases. J Mol Biol 375: 1293-1305.

10. Tsujibo H, Miyamoto K, Kuda T, Minami K, Sakamoto T, et al. (1992) Purification, properties, and partial amino-acid-sequences of thermostable xylanases from *Streptomyces-thermoviolaceus* Opc-520. Appl Environ Microb 58: 371-375.

11. Ko CH, Tsai CH, Tu J, Lee HY, Ku LT, et al. (2010) Molecular cloning and characterization of a novel thermostable xylanase from *Paenibacillus campinasensis* BL11. Process Biochem 45: 1638-1644.

12. Kui H, Luo HY, Shi PJ, Bai YG, Yuan TZ, et al. (2010) Gene cloning, expression, and characterization of a thermostable xylanase from *Nesterenkonia* xinjiangensis CCTCC AA001025. Appl Biochem Biotech 162: 953-965.

13. Viet DN, Kamio Y, Abe N, Kaneko J, Izaki K (1991) Purification and properties of beta-1,4-xylanase from *Aeromonas-caviae* W-61. Appl Environ Microb 57: 445-449.

14. Yeasmin S, Kim CH, Park HJ, Sheikh MI, Lee JY, et al. (2011) Cell surface display of cellulase activity-free xylanase enzyme on *Saccharomyces* *cerevisiae* EBY100. Appl Biochem Biotech 164: 294-304.

15. Belien T, Van Campenhout S, Van Acker M, Volckaert G (2005) Cloning and characterization of two endoxylanases from the cereal phytopathogen *Fusarium graminearum* and their inhibition profile against endoxylanase inhibitors from wheat. Biochem Bioph Res Co 327: 407-414.

16. Yoon KH (2009) Cloning of a *Bacillus subtilis* AMX-4 xylanase gene and characterization of the gene product. J Microbiol Biotechnol 19: 1514-1519.

17. Qu W, Shao W (2011) Cloning, expression and characterization of glycoside hydrolase family 11 endoxylanase from *Bacillus pumilus* ARA. Biotechnol Lett 33: 1407-1416.

18. Amaya-Delgado L, Mejia-Castillo T, Santiago-Hernandez A, Vega-Estrada J, Amelia FG, et al. (2010) Cloning and expression of a novel, moderately thermostable xylanase-encoding gene (Cflxyn11A) from *Cellulomonas flavigena*. Bioresour Technol 101: 5539-5545.

19. Devillard E, Newbold CJ, Scott KP, Forano E, Wallace RJ, et al. (1999) A xylanase produced by the rumen anaerobic protozoan *Polyplastron multivesiculatum* shows close sequence similarity to family 11 xylanases from Gram-positive bacteria. FEMS Microbiol Lett 181: 145-152.

20. Nakamura M, Nagamine T, Takenaka A, Aminov RI, Ogata K, et al. (2002) Molecular cloning, nucleotide sequence and characteristics of a xylanase gene (xynA) from *Ruminococcus albus* 7. Anim Sci J 73 6.

21. Ruller R, Rosa JC, Faca VM, Greene LJ, Ward RJ (2006) Efficient constitutive expression of *Bacillus subtilis* xylanase A in *Escherichia coli* DH5alpha under the control of the *Bacillus* BsXA promoter. Biotechnol Appl Biochem 43: 9-15.

22. Lu P, Feng MG, Li WF, Hu CX (2006) Construction and characterization of a bifunctional fusion enzyme of *Bacillus*-sourced beta-glucanase and xylanase expressed in *Escherichia coli*. FEMS Microbiol Lett 261: 224-230.

23. Lee CC, Kibblewhite-Accinelli RE, Smith MR, Wagschal K, Orts WJ, et al. (2008) Cloning of *Bacillus licheniformis* xylanase gene and characterization of recombinant enzyme. Curr Microbiol 57: 301-305.

24. Gallardo O, Diaz P, Pastor FI (2004) Cloning and characterization of xylanase A from the strain *Bacillus* sp. BP-7: comparison with alkaline pI-low molecular weight xylanases of family 11. Curr Microbiol 48: 276-279.

25. Lee TH, Lim PO, Lee YE (2007) Cloning, characterization, and expression of xylanase A gene from *Paenibacillus* sp. DG-22 in *Escherichia coli*. J Microbiol Biotechnol 17: 29-36.

26. Jeya M, Thiagarajan S, Lee JK, Gunasekaran P (2009) Cloning and expression of GH11 xylanase gene from *Aspergillus fumigatus* MKU1 in Pichia pastoris. J Biosci Bioeng 108: 24-29.

27. Georis J, Giannotta F, De Buyl E, Granier B, Frere J (2000) Purification and properties of three endo-beta-1,4-xylanases produced by *Streptomyces* sp. strain S38 which differ in their ability to enhance the bleaching of kraft pulps*(2). Enzyme Microb Technol 26: 178-186.

28. Kimura T, Suzuki H, Furuhashi H, Aburatani T, Morimoto K, et al. (2000) Molecular cloning, overexpression, and purification of a major xylanase from *Aspergillus oryzae*. Biosci Biotechnol Biochem 64: 2734-2738.

29. Jalal A, Rashid N, Rasool N, Akhtar M (2009) Gene cloning and characterization of a xylanase from a newly isolated *Bacillus subtilis* strain R5. J Biosci Bioeng 107: 360-365.

30. Brennan Y, Callen WN, Christoffersen L, Dupree P, Goubet F, et al. (2004) Unusual microbial xylanases from insect guts. Appl Environ Microbiol 70: 3609-3617.

31. Cazemier AE, Verdoes JC, van Ooyen AJ, Op den Camp HJ (1999) Molecular and biochemical characterization of two xylanase-encoding genes from *Cellulomonas pachnodae*. Appl Environ Microbiol 65: 4099-4107.

32. Liu JR, Duan CH, Zhao X, Tzen JT, Cheng KJ, et al. (2008) Cloning of a rumen fungal xylanase gene and purification of the recombinant enzyme via artificial oil bodies. Appl Microbiol Biotechnol 79: 225-233.

33. Zhang G, Rao B, Ye J, Ma L, Zhang X (2008) Molecular cloning and heterologous expression of a new xylanase gene from *Verticillium dahliae*. Acta Microbiologica Sinica 48: 765-771.

34. Zhou P, Zhu H, Yan Q, Katrolia P, Jiang Z (2011) Purification and properties of a psychrotrophic *Trichoderma* sp. xylanase and its gene sequence. Appl Biochem Biotechnol 164: 944-956.

35. Zhang GM, Huang J, Huang GR, Ma LX, Zhang XE (2007) Molecular cloning and heterologous expression of a new xylanase gene from *Plectosphaerella cucumerina*. Appl Microbiol Biotechnol 74: 339-346.

36. McIntosh LP, Hand G, Johnson PE, Joshi MD, Korner M, et al. (1996) The p*K*_a_ of the general acid/base carboxyl group of a glycosidase cycles during catalysis: a 13C-NMR study of *Bacillus circulans* xylanase. Biochemistry 35: 9958-9966.

37. Lee JM, Shin JW, Nam JK, Choi JY, Jeong CS, et al. (2009) Molecular cloning and expression of the *Trichoderma harzianum* C4 endo-beta-1,4-xylanase gene in *Saccharomyces cerevisiae*. J Microbiol Biotechn 19: 823-828.

38. Torronen A, Harkki A, Rouvinen J (1994) Three-dimensional structure of endo-1,4-beta-xylanase II from *Trichoderma reesei*: two conformational states in the active site. EMBO J 13: 2493-2501.

39. Yang HM, Yao B, Luo HY, et al. (2005) Hydrophobic interaction between beta-sheet B1 and B2 in xylanase XYNB influencing the enzyme thermostability. Chin J Biotech 21: 414–419.

40. Furniss CSM, Belshaw NJ, Alcocer MJC, Williamson G, Elliott GO, et al. (2002) A family 11 xylanase from *Penicillium funiculosum* is strongly inhibited by three wheat xylanase inhibitors. BBA-Proteins Proteom 1598: 24-29.

41. Matteotti C, Bauwens J, Brasseur C, Tarayre C, Thonart P, et al. (2012) Identification and characterization of a new xylanase from Gram-positive bacteria isolated from termite gut (*Reticulitermes santonensis*). Protein Expres Purif 83: 117-127.

42. Krisana A, Rutchadaporn S, Jarupan G, Lily E, Sutipa T, et al. (2005) Endo-1,4-beta-xylanase B from *Aspergillus cf. niger* BCC14405 isolated in Thailand: purification, characterization and gene isolation. J Biochem Mol Biol 38: 17-23.

43. Tanaka H, Nakamura T, Hayashi S, Ohta K (2005) Purification and properties of an extracellular endo-1,4-beta-xylanase from *Penicillium citrinum* and characterization of the encoding gene. J Biosci Bioeng 100: 623-630.

44. Wakiyama M, Yoshihara K, Hayashi S, Ohta K (2010) An extracellular endo-1,4-beta-xylanase from *Aspergillus japonicus*: Purification, properties, and characterization of the encoding gene. J Biosci Bioeng 109: 227-229.

45. Deng P, Li DF, Cao YH, Lu WQ, Wang CL (2006) Cloning of a gene encoding an acidophilic endo-beta-1,4-xylanase obtained from *Aspergillus niger* CGMCC1067 and constitutive expression in *Pichia pastoris*. Enzyme Microb Tech 39: 1096-1102.

46. Li YH, Zhang B, Chen X, Chen YQ, Cao YH (2010) Improvement of *Aspergillus sulphureus* endo-beta-1,4-xylanase expression in *Pichia pastoris* by codon optimization and analysis of the enzymic characterization. Appl Biochem Biotech 160: 1321-1331.

47. Korona B, Korona D, Bielecki S (2006) Efficient expression and secretion of two co-produced xylanases from *Aspergillus niger* in *Pichia pastoris* directed by their native signal peptides and the *Saccharomyces cerevisiae* alpha-mating factor. Enzyme Microb Tech 39: 683-689.

48. Brutus A, Reca IB, Herga S, Mattei B, Puigserver A, et al. (2005) A family 11 xylanase from the pathogen *Botrytis cinerea* is inhibited by plant endoxylanase inhibitors XIP-I and TAXI-I. Biochem Bioph Res Co 337: 160-166.

49. Zhou C, Bai J, Deng S, Wang J, Zhu J, et al. (2008) Cloning of a xylanase gene from *Aspergillus usamii* and its expression in *Escherichia coli*. Bioresour Technol 99: 831-838.

50. Lee CC, Wong DW, Robertson GH (2005) Cloning and characterization of the xyn11A gene from *Lentinula edodes*. Protein J 24: 21-26.

51. Liu W, Shi P, Chen Q, Yang P, Wang G, et al. (2010) Gene cloning, overexpression, and characterization of a xylanase from *Penicillium* sp. CGMCC 1669. Appl Biochem Biotechnol 162: 1-12.

52. Decelle B, Tsang A, Storms RK (2004) Cloning, functional expression and characterization of three *Phanerochaete chrysosporium* endo-1,4-beta-xylanases. Curr Genet 46: 166-175.

53. Furniss CSM, Williamson G, Kroon PA (2005) The substrate specificity and susceptibility to wheat inhibitor proteins of *Penicillium funiculosum* xylanases from a commercial enzyme preparation. J Sci Food Agr 85: 574-582.

54. Luttig M, Pretorius IS, vanZyl WH (1997) Cloning of two beta-xylanase-encoding genes from *Aspergillus niger* and their expression in *Saccharomyces cerevisiae*. Biotechnol Lett 19: 411-415.

55. Belancic A, Scarpa J, Peirano A, Diaz R, Steiner J, et al. (1995) *Penicillium purpurogenum* produces several xylanases: purification and properties of two of the enzymes. J Biotechnol 41: 71-79.

56. Torronen A, Rouvinen J (1995) Structural comparison of two major endo-1,4-xylanases from *Trichoderma reesei*. Biochemistry 34: 847-856.

57. Al Balaa B, Wouters J, Dogne S, Rossini C, Schaus JM, et al. (2006) Identification, cloning, and expression of the *Scytalidium acidophilum* XYL1 gene encoding for an acidophilic xylanase. Biosci Biotechnol Biochem 70: 269-272.

58. Parachin NS, Siqueira S, de Faria FP, Torres FAG, de Moraes LMP (2009) Xylanases from *Cryptococcus flavus* isolate I-11: Enzymatic profile, isolation and heterologous expression of CfXYN1 in *Saccharomyces cerevisiae*. J Mol Catal B-Enzym 59: 52-57.

59. Driss D, Bhiri F, Ghorbel R, Chaabouni SE (2012) Cloning and constitutive expression of His-tagged xylanase GH 11 from *Penicillium occitanis* Pol6 in *Pichia pastoris* X33: Purification and characterization. Protein Expres Purif 83: 8-14.

60. Krengel U, Dijkstra BW (1996) Three-dimensional structure of endo-1,4-beta-xylanase I from *Aspergillus niger*: Molecular basis for its low pH optimum. J Mol Biol 263: 70-78.

61. Ohta K, Moriyama S, Tanaka H, Shige T, Akimoto H (2001) Purification and characterization of an acidophilic xylanase from *Aureobasidium pullulans* *var. melanigenum* and sequence analysis of the encoding gene. J Biosci Bioeng 92: 262-270.

62. Iefuji H, Chino M, Kato M, Iimura Y (1996) Acid xylanase from yeast *Cryptococcus* sp. S-2: Purification, characterization, cloning, and sequencing. Biosci Biotech Bioch 60: 1331-1338.

63. Kimura T, Ito J, Kawano A, Makino T, Kondo H, et al. (2000) Purification, characterization, and molecular cloning of acidophilic xylanase from *Penicillium* sp 40. Biosci Biotech Bioch 64: 1230-1237.

64. Ito K, Ogasawara H, Sugimoto T, Ishikawa T (1992) Purification and properties of acid stable xylanases from *Aspergillus kawachii*. Biosci Biotech Bioch 56: 547-550.
