# Supplementary material for: Structural Insight into and Mutational Analysis of Family 11 Xylanases: Implications for Mechanisms of Higher pH Catalytic Adaptation
Source: PLoS One. 2015 Jul 10;10(7):e0132834. doi: 10.1371/journal.pone.0132834 (PMC4498622; doi:10.1371/journal.pone.0132834)
Supplement: S2 Table — (DOC) [file pone.0132834.s005.doc]

Table S2 Molecular feature comparison of family 11 xylanases grouped into different phylogenetic clusters (Cluster A and Cluster B)

| Parameters | Mean | | t-value | P |
| --- | --- | --- | --- | --- |
|  | Cluster A | Cluster B |  |  |
| Optimum pH | 7.316 | 5.049 | 4.509 | 0.000 |
| Ala (%) | 4.558 | 5.552 | -2.632 | 0.012 |
| Arg (%) | 3.437 | 3.062 | 1.356 | 0.180 |
| Asn (%) | 7.681 | 7.987 | -0.566 | 0.573 |
| Asp (%) | 4.909 | 3.614 | 3.219 | 0.004 |
| Cys (%) | 0.811 | 0.386 | 2.582 | 0.012 |
| Gln (%) | 4.456 | 3.575 | 2.558 | 0.013 |
| Glu (%) | 4.573 | 2.835 | 4.909 | 0.000 |
| Gly (%) | 12.204 | 13.232 | -2.650 | 0.010 |
| His (%) | 1.410 | 1.339 | 0.384 | 0.702 |
| Ile (%) | 5.306 | 3.310 | 9.983 | 0.000 |
| Leu (%) | 4.288 | 2.882 | 6.505 | 0.000 |
| Lys (%) | 5.282 | 1.905 | 9.757 | 0.000 |
| Met (%) | 2.011 | 0.973 | 6.573 | 0.000 |
| Phe (%) | 3.936 | 3.403 | 1.892 | 0.063 |
| Pro (%) | 2.928 | 3.154 | -1.118 | 0.268 |
| Ser (%) | 7.992 | 11.822 | -7.776 | 0.000 |
| Thr (%) | 9.306 | 11.584 | -4.048 | 0.000 |
| Trp (%) | 3.304 | 3.635 | -1.028 | 0. 308 |
| Tyr (%) | 6.461 | 8.396 | -6.916 | 0.000 |
| Val (%) | 5.328 | 7.352 | -5.285 | 0.000 |
| Charged residues（DERK）(%) | 18.053 | 11.415 | 10.496 | 0.000 |
| Acidic (DE) (%) | 9.302 | 6.449 | 5.249 | 0.000 |
| Basic (RK) (%) | 8.778 | 4.967 | 8.189 | 0.000 |
| charged residue ratio (-ve/+ve) | 1.073 | 1.665 | -3.059 | 0.003 |
| Polar (NCQSTY) (%) | 36.706 | 43.751 | -9.353 | 0.000 |
| Hydrophobic (AILFWV) (%) | 26.719 | 26.092 | 0.988 | 0.327 |
