# Supplementary material for: Structural Insight into and Mutational Analysis of Family 11 Xylanases: Implications for Mechanisms of Higher pH Catalytic Adaptation
Source: PLoS One. 2015 Jul 10;10(7):e0132834. doi: 10.1371/journal.pone.0132834 (PMC4498622; doi:10.1371/journal.pone.0132834)
Supplement: S3 Table — (DOC) [file pone.0132834.s006.doc]

Table S3 Molecular feature comparison of family 11 xylanase grouped into different phylogenetic clusters (Cluster B1 and Cluster B2)

| Parameters | Mean | | t-value | P |
| --- | --- | --- | --- | --- |
|  | Cluster B1 | Cluster B2 |  |  |
| Optimum pH | 5.725 | 3.214 | 7.675 | 0.000 |
| Ala (%) | 5.085 | 6.917 | -3.933 | 0.000 |
| Arg (%) | 3.339 | 2.251 | 4.768 | 0.000 |
| Asn (%) | 8.173 | 7.440 | 1.157 | 0.253 |
| Asp (%) | 3.584 | 3.702 | -0.335 | 0.739 |
| Cys (%) | 0.193 | 0.951 | -5.871 | 0.000 |
| Gln (%) | 3.302 | 4.372 | -2.676 | 0.010 |
| Glu (%) | 2.649 | 3.380 | -1.857 | 0.069 |
| Gly (%) | 13.853 | 11.417 | 5.772 | 0.000 |
| His (%) | 1.362 | 1.274 | 0.489 | 0.627 |
| Ile (%) | 3.357 | 3.174 | 0.745 | 0.460 |
| Leu (%) | 2.853 | 2.965 | -0.582 | 0.563 |
| Lys (%) | 2.340 | 0.631 | 6.027 | 0.000 |
| Met (%) | 0.954 | 1.029 | -0.420 | 0.677 |
| Phe (%) | 3.034 | 4.480 | -5.372 | 0.000 |
| Pro (%) | 3.218 | 2.965 | 1.054 | 0.297 |
| Ser (%) | 11.060 | 14.050 | -4.325 | 0.000 |
| Thr (%) | 12.150 | 9.928 | 3.993 | 0.000 |
| Trp (%) | 3.993 | 2.587 | 3.840 | 0.000 |
| Tyr (%) | 8.496 | 8.104 | 0.692 | 0.350 |
| Val (%) | 7.000 | 8.380 | -3.896 | 0.001 |
| Charged residues (DERK) (%) | 11.912 | 9.964 | 3.527 | 0.001 |
| Acidic (DE) (%) | 6.233 | 7.082 | -1.480 | 0.145 |
| Basic (RK) (%) | 5.680 | 2.883 | 6.944 | 0.000 |
| charged residue ratio (-ve/+ve) | 1.185 | 3.067 | -5.824 | 0.000 |
| Polar (NCQSTY) (%) | 43.376 | 44.845 | -1.981 | 0.053 |
| Hydrophobic (AILFWV) (%) | 25.324 | 28.504 | -5.263 | 0.000 |
